# CKAP4 Regulates ERS‐Induced Apoptosis in Osteoclasts Through FOXO3 in Periodontitis

**DOI:** 10.1111/cpr.70208

**Published:** 2026-04-13

**Authors:** Jiayu Cheng, Dihao Tao, Wenzhe Wang, Huiyu Wang, Hanzhe Wang, Yufan Liang, Jia Guo, Wenhao Liu, Yiming Wang, Jianhua Yang, Xiaoning He, Ce Shan, Bei Li

**Affiliations:** ^1^ State Key Laboratory of Oral & Maxillofacial Reconstruction and Regeneration, National Clinical Research Center for Oral Diseases. Shaanxi International Joint Research Center for Oral Diseases, Center for Tissue Engineering, School of Stomatology The Fourth Military Medical University Xi'an Shaanxi China

**Keywords:** apoptosis, CKAP4, endoplasmic reticulum stress, FOXO3, osteoclast, periodontitis

## Abstract

Periodontitis is a prevalent oral disease characterised by chronic inflammation and irreversible alveolar bone loss. Osteoclasts (OCs) are the key cells mediating bone resorption and their excessive formation disrupts bone homeostasis. Since apoptosis of OCs normally restrains this process, its failure can sustain bone loss. However, its role in periodontitis remains unclear. Analysis of single‐cell RNA sequencing (scRNA‐seq) data retrieved from the Gene Expression Omnibus (GEO) database revealed impaired OC apoptosis in the periodontal tissues of individuals suffering from periodontitis. Notably, we observed a reduction in cytoskeleton‐associated protein 4 (CKAP4) expression correlating with decreased OC apoptosis. Further investigations using *Ckap4* knockout mice confirmed that CKAP4 promotes OC apoptosis through the activation of forkhead box O3 (FOXO3)‐induced endoplasmic reticulum stress (ERS). CKAP4 regulates OC apoptosis to maintain periodontal homeostasis and its downregulation in periodontitis promotes pathological bone resorption. This study elucidates CKAP4‐mediated apoptotic pathways in OCs, providing mechanistic insight and potential therapeutic strategies to restore OC balance and prevent bone loss.

## Graphical abstract

CKAP4 promotes the transcription of PERK via FOXO3 in OCs, thereby facilitating ER stress‐induced apoptosis. In periodontitis, the reduction of CKAP4 in OCs leads to decreased OC apoptosis, which exacerbates alveolar bone resorption.

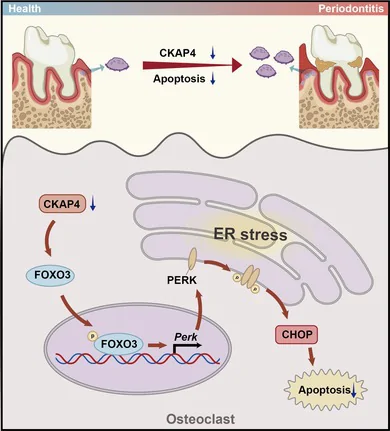

## 1 Introduction

Periodontitis, a highly prevalent oral disease, impacts approximately 62% of the global adults and stands as a major contributor to tooth loss in middle‐aged and elderly people [1]. The level of alveolar bone resorption stands as a crucial determinant that impacts the course and prognosis of periodontitis [2]. However, current treatment modalities fail to effectively restore alveolar bone volume, which remains a pressing challenge to be addressed in periodontitis management [3].

Osteoclasts (OCs) are terminally differentiated cells that serve as the key effector cells mediating alveolar bone resorption in periodontitis [4]. The excessive number of OCs disrupts the homeostasis of alveolar bone, resulting in sustained bone resorption [5]. To date, the majority of studies investigating the accumulation of OCs have focused on the regulatory machinery of cellular differentiation. For instance, several groups reported that enhanced differentiation of precursor OCs into mature OCs is activated by immune cells in response to dental plaque stimulation [6]. Yet clinically, though eliminating irritants alleviates inflammation, alveolar bone volume cannot be effectively recovered, which greatly complicates treatment outcomes [7]. This indicates that the ‘inflammation‐differentiation’ alone cannot fully explain OCs' accumulation, and there may be an unrecognized mechanism that sustains OCs numbers even when inflammation (and thus differentiation) is suppressed.

From a cellular homeostasis perspective, the number of OCs is determined by the dynamic balance between cell production (differentiation) and cell clearance (apoptosis). In healthy periodontal tissues, the rate of OC apoptosis is balanced with the rate of precursor OC differentiation, ensuring that bone resorption is countered by bone formation mediated by osteoblasts, thereby maintaining the integrity of the alveolar bone [8]. Studies in rheumatoid arthritis have shown a decreased apoptotic rate of OCs, indicating that insufficient apoptosis contributes to continuous progression of joint inflammation and tissue destruction [9]. However, in periodontitis, which is also an inflammatory bone disease, the status of OC apoptosis remains to be elucidated. Unlike reversible bone loss in acute inflammation, the irreversibility of alveolar bone destruction in periodontitis is thought to originate from the persistent activation of OCs [10], and OC apoptosis is a key checkpoint that terminates their bone‐eroding activity. Therefore, the apoptotic capacity of OCs may be closely linked to the irreversibility of alveolar bone loss after periodontal therapy.

Cytoskeleton‐associated protein 4 (CKAP4), a Type II transmembrane protein initially identified in the endoplasmic reticulum (ER) [11], has been shown to be involved in multiple cellular processes, such as proliferation [12], migration [13] and apoptosis [14]. In tumour cells, CKAP4 promotes apoptosis by facilitating the recruitment of BAX to the ER and mitochondrial membranes, triggers the release of cytochrome c [14], and regulates ER stress (ERS) [11]. However, its role in OCs, particularly in the context of periodontitis, has not been fully explored.

In this study, we demonstrate that CKAP4 promotes OC apoptosis by activating the ERS and that reduced CKAP4 expression in periodontitis impairs OC apoptosis, thereby exacerbating alveolar bone resorption. These findings yield new understanding of the molecular mechanisms underlying periodontitis‐related bone loss and identify CKAP4 as a potential candidate for therapeutic intervention.

## 2 Materials and Methods

### 2.1 Animals

C57BL/6J (stock no. 000664) mouse strains were obtained from Animal Center of the Fourth Military Medical University. *Ckap4* global‐knockout (*Ckap4* ^*−/−*^, stock no. S‐KO‐05148), *Ctsk*‐iCre (stock no. C001366) and *Ckap4* ^*flox/flox*^ (referred to as *Ckap4* ^*fl/fl*^ herein, stock no. NM‐CKO‐200259) mouse strains were generated by Cyagen Biosciences Inc. (Suzhou, China). *Ctsk*‐iCre mice were crossed with *Ckap4* ^*fl/fl*^ mice to generate *Ctsk*‐cre::*Ckap4* ^*flox/flox*^ (conditional deletion of *Ckap4* in *Ctsk* lineage cells, referred to as *Ckap4* ^*ΔOC*^ herein). Mouse genotypes were identified via polymerase chain reaction (primers: Table S1) amplification of genomic DNA extracted from mouse tails. Male mice at 6–8 weeks of age were utilised for all experiments.

### 2.2 Animal Model of Periodontitis

Ligature‐induced periodontitis was constructed in 6–8‐week‐old male mice by ligation of 5‐0 silk sutures around the cervical area of maxillary second molars, with knots fastened on the buccal surface. The ligatures were retained for 7 or 14 days, while age‐matched mice without ligature placement served as blank controls. At the conclusion of each experiment, the mice were euthanized and the maxillae were dissected and fixed in 4% paraformaldehyde (PFA; Sigma‐Aldrich, USA) at 4°C overnight. Samples were scanned using a micro‐CT system (Siemens Inveon; Munich, Germany) with parameters set at 18 μm resolution, 50 kV and 80 μA. The reconstructed data were analysed via VGStudio MAX software (Volume Graphics, Germany), and the cementoenamel junction‐alveolar bone crest (CEJ‐ABC) distance, bone volume/tissue volume (BV/TV) and trabecular number (Tb.N) were quantified on the mesial and distal aspects of the maxillary second molars.

### 2.3 Single‐Cell RNA‐Seq Data Analysis

Healthy/periodontitis periodontal scRNA‐seq datasets (GSE171213, GSE152042, GSE161267) were integrated via Harmony algorithm. Cell clusters were annotated by differential gene expression. We defined the OC population from single‐cell transcriptomic data by selecting cells with high expression of OC‐specific markers (*CTSK* and *TRAP*) while excluding contaminating lineages (fibroblasts, macrophages, lymphocytes). To identify apoptosis‐associated genes, we employed two independent classification strategies: (1) stratifying OCs into high‐*BAX* (top 30%) versus low‐*BAX* (bottom 30%) subgroups and (2) stratifying into high‐*CYCS* (top 30%) versus low‐*CYCS* (bottom 30%) subgroups. Differentially expressed genes (DEGs) analysis between respective subgroups (|log_2_ FC| > 1, adjusted *p* < 0.01) revealed *CKAP4*, *PMAIP1* and *UBE2T* as the only overlapping genes among the Top 10 significantly altered genes from each comparison.

### 2.4 OCs Extraction and Induction

OC differentiation was induced in BMDMs (bone marrow‐derived macrophages) obtained from the bilateral femurs and tibias of mice. Bone marrow‐derived cells were cultured in alpha minimum essential medium (α‐MEM) with 10% foetal bovine serum (FBS; Tianhang, Zhejiang, China) and 30 ng/mL murine macrophage colony‐stimulating factor (M‐CSF; PeproTech, USA). Nonadherent BMDMs were collected, rinsed and re‐plated at a cell density of 1 × 10^6^ cells per millilitre in 24‐well plates with osteoclastogenic medium (containing 30 ng/mL M‐CSF and 50 ng/mL receptor activator of nuclear factor‐κB (NF‐κB) ligand [RANKL]; R&D Systems, USA). The cellular morphology was monitored for the formation of large multinucleated cells.

### 2.5 Western Blot (WB)

Total protein extracts were prepared using radio‐immunoprecipitation assay (RIPA) buffer and centrifuged at 12,000*g* for 10 min. Protein concentration was quantified using the bicinchoninic acid (BCA) protein assay kit. Equal protein samples were resolved by sodium dodecyl sulphate‐polyacrylamide gel electrophoresis (SDS‐PAGE) gels (Epizyme Biotech, China) and electrophoretically transferred to polyvinylidene fluoride (PVDF) membranes. Membranes were blocked for 2 h in 5% bovine serum albumin (BSA, Sigma‐Aldrich, USA) dissolved in PBST. Subsequently, the membranes were incubated with primary antibodies (detailed in Table S2) at 4°C overnight. Following phosphate‐buffered saline with Tween 20 (PBST) washes, the membranes were incubated with peroxidase‐conjugated secondary antibodies (diluted 1:10,000) for 2 h at room temperature. Bands were detected using an enhanced chemiluminescence (ECL) kit (Amersham Biosciences, USA) and captured via the Western‐Light chemiluminescence detection system. Uncropped pictures of the WB bands presented in this study are all shown in Figures S6 and S7.

### 2.6 Quantitative Real‐Time Polymerase Chain Reaction (qRT‐PCR)

Total RNA was isolated from cells using TRIzol Reagent (Invitrogen, USA), and reverse transcribed into complementary DNA with the PrimeScript RT Reagent Kit (Takara, Japan). qRT‐PCR was conducted with the SYBR Premix Ex Taq II Kit (Takara, Japan) on a real‐time PCR instrument (Bio‐Rad, USA). The relative expression level of each gene was calculated via the 2^−ΔΔ*C*t^ method, with β‐actin serving as the internal reference gene for normaliation. All assays were performed in triplicate and repeated in at least three independent experiments. Table S3 contains the sequences of all primers used in the study.

### 2.7 RNA‐Seq and Data Analysis

RNA quantity and purity were assessed via Bioanalyzer 2100 (Agilent). Libraries were constructed with VAHTS Universal V6 Kit, sequenced on Illumina NovaSeq 6000 (150 bp paired‐end). Raw reads were filtered via fastp, aligned to reference genome via HISAT2. Gene expression levels were quantified as fragments per kilobase of exon per million reads mapped (FPKM) via StringTie and Ballgown. A heatmap was generated with pheatmap to visualise intersample differences in gene expression. DEGs (|FC| > 2, *p* < 0.05) were annotated to kyoto encyclopaedia of genes and genomes (KEGG) pathways.

### 2.8 Lentivirus (LV) Transduction

Recombinant LVs for *Ckap4* and *Foxo3* overexpression or silencing were acquired from Vigene Biosciences (Vigene Bio, China). Table S4 contains the sequences of all shRNAs used in the study. Stable OCs overexpressing *Ckap4* (*oeCkap4*) and *Foxo3* (*oeFoxo3*) or silencing *Foxo3* (*shFoxo3*) and their corresponding negative controls (*oeCkap4*‐Ctrl, *oeFoxo3*‐Ctrl and *shFoxo3*‐Ctrl) were generated from BMDMs one day after isolation, followed by differentiation induction with M‐CSF and RANKL and final confirmation of viral transduction efficiency.

### 2.9 Statistical Analysis

All analyses were conducted using data from no fewer than three independent biological replicates. Statistical analysis was performed with GraphPad Prism 8.0 software. Comparisons between two groups were carried out via two‐tailed Student's *t*‐test, while comparisons among multiple groups were analysed using one‐way analysis of variance (ANOVA) combined with Tukey's or Dunnett's post hoc test. A difference between groups was deemed statistically significant at *p < 0.05, highly significant at **p < 0.01 and extremely significant at ***p < 0.001.

## 3 Results

### 3.1 Periodontitis Exhibits Diminished OC Apoptosis and Decreased CKAP4

**FIGURE 1:**
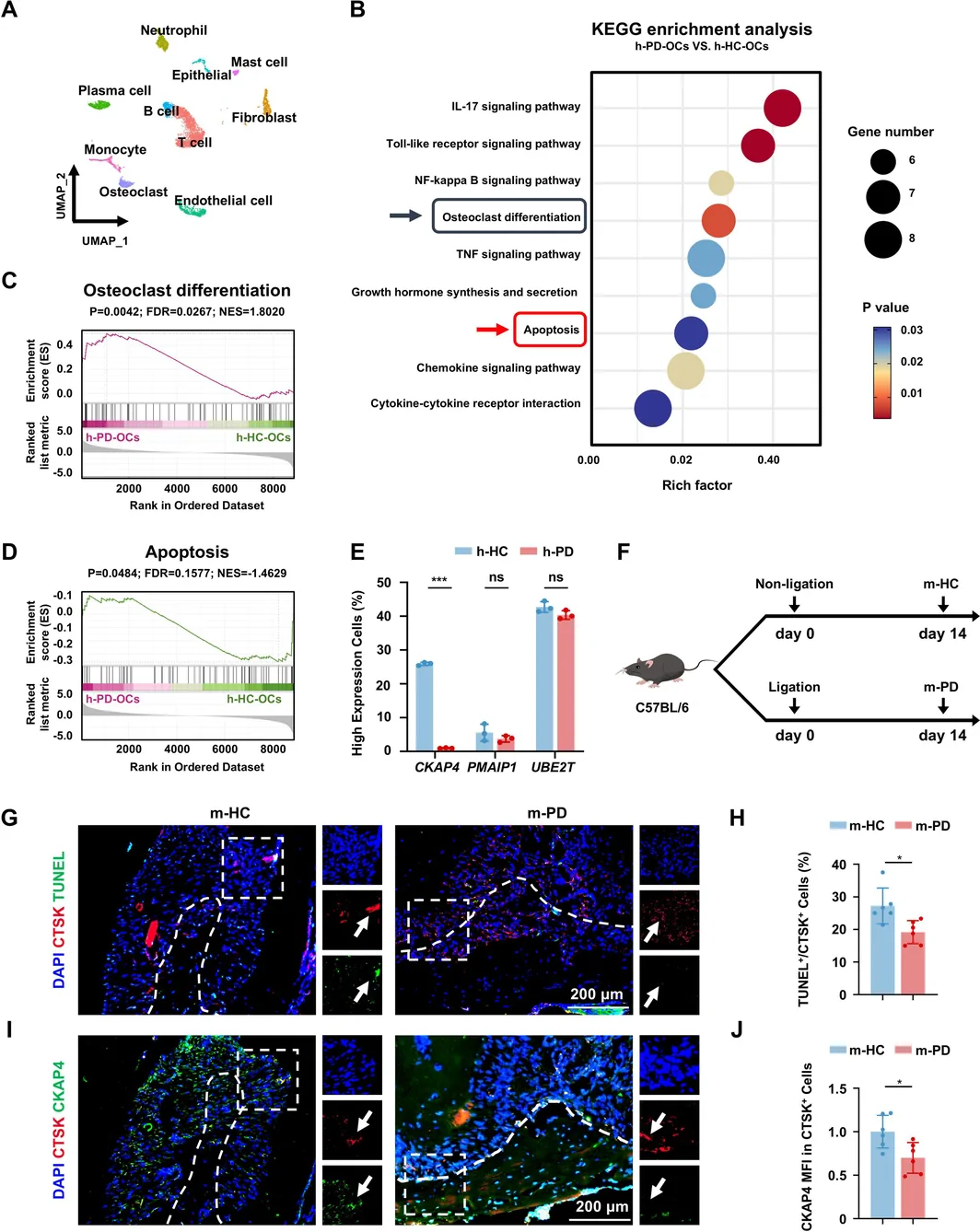
Periodontitis exhibits diminished OC apoptosis and decreased CKAP4. (A) UMAP representation of RNA‐seq data (GSE171213, GSE152042, GSE161267) from periodontal tissues of patients in h‐PD and h‐HC groups, with OCs labelled. (B) KEGG enrichment scatter plot comparing h‐PD and h‐HC samples, highlighting pathways related to OC differentiation (black arrow) and apoptosis (red arrow). Enrichment results for OC differentiation (C) and apoptosis (D) pathways via GSEA. (E) The proportion of OCs with high *CKAP4*, *PMAIP1* and *UBE2T* expression from h‐HC and h‐PD samples (*n* = 3). (F) Experimental design for (G–J). Silk ligation was performed on Day 0, and the mice were sacrificed on day 14 (m‐PD). Mice without ligation served as the healthy control (m‐HC). (G) Immunofluorescence images of CTSK (red), TUNEL (green) and DAPI (blue) in m‐HC and m‐PD periodontal tissues (dashed boxes: double‐positive regions; white arrows: Double‐positive cells). Scale bar: 200 μm. (H) The proportion of TUNEL^+^/CTSK^+^ double‐positive cells (*n* = 6). (I) Immunofluorescence images of CTSK (red), CKAP4 (green) and DAPI (blue) in m‐HC and m‐PD periodontal tissues (dashed boxes: double‐positive regions; white arrows: double‐positive cells). Scale bar: 200 μm. (J) CKAP4 mean fluorescence intensity (MFI) in CTSK‐positive (CTSK^+^) cells, presented as fold change over m‐HC (*n* = 6). Results are shown as mean ± SD. Each dot indicates an individual sample or mouse. Student's *t*‐test (E, H and J) was performed. ***p < 0.001; **p < 0.01; *p < 0.05; ns, not significant.

The number of OCs is a key indicator of the progression and severity of periodontitis. Severe bone resorption mediated by OCs induces alveolar bone loss, which ultimately leads to tooth loss [15]. To investigate mechanisms of OC accumulation in periodontitis, we obtained GEO single‐cell RNA‐seq datasets of periodontal tissues from periodontitis patients (h‐PD) and healthy controls (h‐HC) (Figure 1A and S1A,B). OCs were identified by typical markers *CTSK* (cathepsin K) and *TRAP* (Figure S1C,D). We performed KEGG analysis and GSEA (gene set enrichment analysis) on two groups of OCs. In OCs from h‐PD (h‐PD‐OCs), the OC differentiation pathway, including the tumour necrosis factor (TNF) signalling pathway and the NF‐κB signalling pathway that mediate OC differentiation [16],was significantly enriched and showed marked upregulation (Figure 1B,C). Notably, the apoptosis pathway in h‐PD‐OCs was substantially impaired (Figure 1B,D), indicating OC accumulation in periodontitis stems from both accelerated differentiation and reduced apoptosis.

To identify OC apoptosis regulators, we classified h‐PD‐OCs into apoptotic OCs and normal OCs based on BCL2‐associated X protein (*BAX*) and cytochrome c (*CYCS*) [17], respectively. We obtained the top 10 most significantly DEGs, among which *CKAP4*, phorbol‐12‐myristate‐13‐acetate‐induced protein 1 (*PMAIP1*) and ubiquitin conjugating enzyme E2 T (*UBE2T*) were the only overlapping genes (Fig. S1E–G). However, the OC subpopulation with high *CKAP4* expression was significantly reduced in h‐PD, while no significant differences were detected in the subsets with high *PMAIP1* or *UBE2T* expression (Figure 1E). These results indicate that *CKAP4*, *PMAIP1* and *UBE2T* may be potential regulators of OC apoptosis during periodontitis, with *CKAP4* likely exerting a more critical regulatory role in h‐PD‐related OC apoptosis.

To verify this hypothesis, we established a murine periodontitis model (m‐PD) via silk ligation, with unligated mice as controls (m‐HC) (Figure 1F). Immunofluorescence staining showed the proportion of TUNEL‐positive (TUNEL^+^) OCs in the m‐PD group significantly decreased (Figure 1G,H), indicating reduced OC apoptosis. Notably, the expression of CKAP4 in OCs was also significantly decreased (Figure 1I,J), consistent with reduced apoptosis. Collectively, these results indicate OC apoptosis is reduced in periodontitis, with CKAP4 potentially regulating OC apoptosis.

### 3.2 Elevated Intracellular CKAP4 Expression Accompanies OC Apoptosis

**FIGURE 2:**
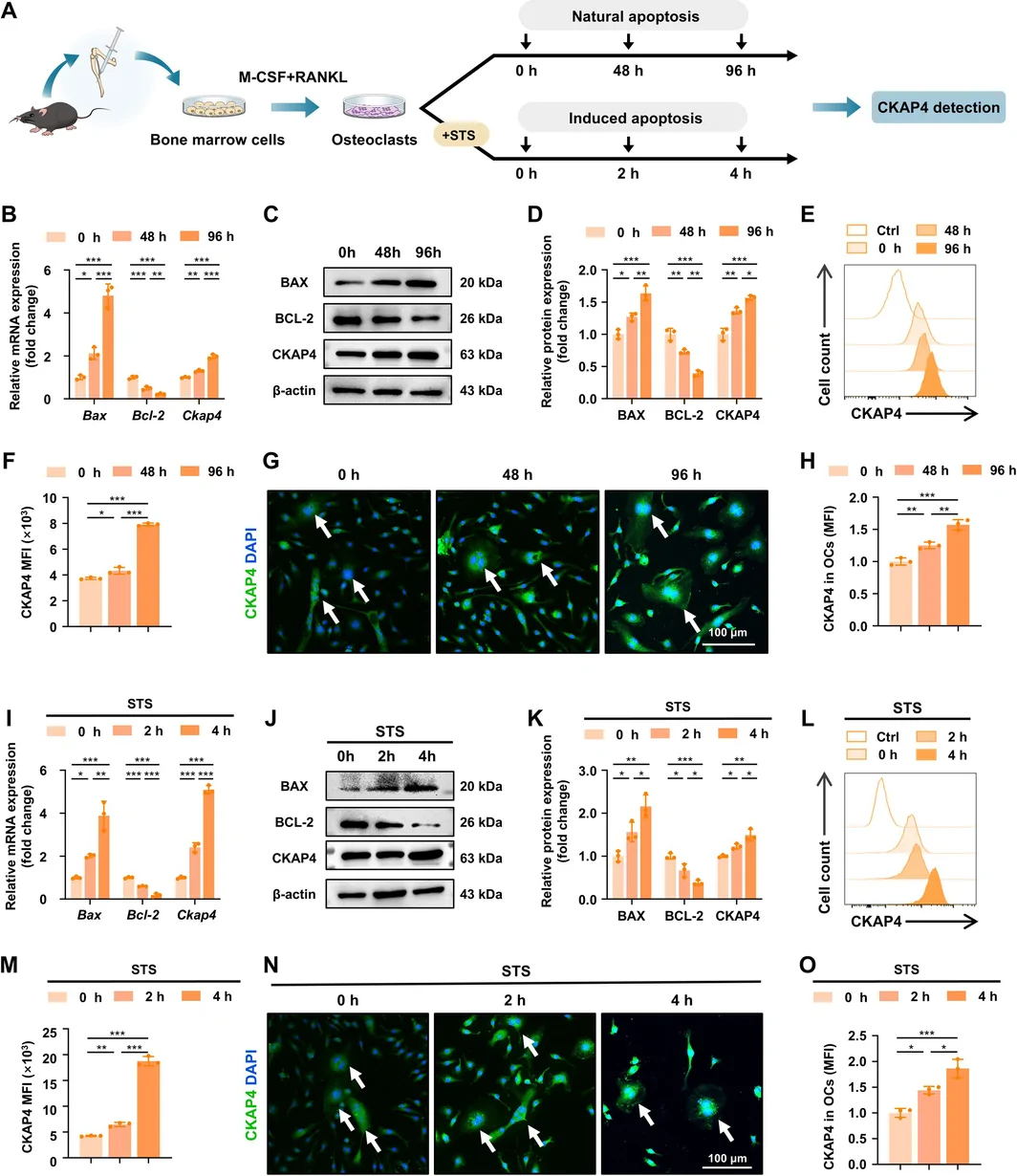
Elevated intracellular CKAP4 expression accompanies OC apoptosis. (A) Experimental design for B‐O. Bone marrow cells were differentiated into OCs with M‐CSF and RANKL. CKAP4 was then detected at 0h, 48 96 h for natural apoptosis and 0, 2 and 4 h for induced apoptosis. (B) *Bax*, *Bcl‐2* and *Ckap4* expression during natural apoptosis was detected by RT‐qPCR, normalised to *β‐actin* and shown as fold change over 0 h (*n* = 3). (C) The expression levels of BAX, BCL‐2 and CKAP4 in OCs during natural apoptosis were confirmed by WB assays. β‐actin was used as a loading control. (D) Quantification of BAX, BCL‐2 and CKAP4 levels was normalised to β‐actin and the results are shown as fold change over the 0 h group (*n* = 3). (E, F) MFI of CKAP4 in OCs during natural apoptosis were examined by flow cytometry (gated on CTSK^+^ cells) (*n* = 3). (G, H) Immunofluorescence staining of CKAP4 (green) and DAPI (blue) in OCs during natural apoptosis. White arrows indicate mature multinucleated OCs. The MFI of CKAP4 in OCs were compared (*n* = 3). Scale bar: 100 μm. (I) *Bax*, *Bcl‐2* and *Ckap4* expression during induced apoptosis was detected by RT‐qPCR, normalised to *β‐actin* and shown as fold change over 0 h (*n* = 3). (J) The expression levels of BAX, BCL‐2 and CKAP4 in OCs during induced apoptosis were confirmed by WB assays. β‐actin was used as a loading control. (K) Quantification of BAX, BCL‐2 and CKAP4 levels was normalised to β‐actin, and the results are shown as fold change over the 0 h group (*n* = 3). (L, M) MFI of CKAP4 in OCs during induced apoptosis were examined by flow cytometry (gated on CTSK^+^ cells) (*n* = 3). (N, O) Immunofluorescence staining of CKAP4 (green) and DAPI (blue) in OCs during induced apoptosis. White arrows indicate mature multinucleated OCs. The MFI of CKAP4 in OCs were compared (*n* = 3). Scale bar: 100 μm. Results are shown as mean ± SD. Each dot indicates an individual sample. One‐way ANOVA with Tukey's post hoc test (B–O) were performed. ***p < 0.001; **p < 0.01; *p < 0.05; ns, not significant.

To clarify the association between CKAP4 and OC apoptosis, we detected CKAP4 expression during OC apoptosis in vitro. To simulate the apoptosis of OCs under natural conditions and with apoptotic stimulants, we detected CKAP4 expression in OCs at 96 h (natural apoptosis) and 4 h post‐staurosporine (STS) induction (induced apoptosis), respectively (Figure 2A). During natural apoptosis, BAX was significantly upregulated at transcriptional and translational levels, whereas B‐cell lymphoma‐2, an anti‐apoptotic protein (BCL‐2), showed a reciprocal decrease (Figure 2B–D). These findings indicate mature OCs gain increased apoptotic sensitivity with prolonged culture. Notably, CKAP4 expression increased continuously in a time‐dependent manner at both levels (Figure 2B–D). Flow cytometry and immunofluorescence validated this trend (Figure 2E–H), suggesting CKAP4 upregulation may contribute to OC apoptosis. Consistent with these findings, STS‐induced OC apoptosis also led to significant BAX upregulation and gradual BCL‐2 reduction (Figure 2I–K), alongside a time‐dependent CKAP4 increase with prolonged STS exposure (Figure 2I–O). Collectively, these data confirm CKAP4 expression correlates with OC apoptosis progression, suggesting CKAP4 upregulation is associated with enhanced OC apoptotic sensitivity.

### 3.3 CKAP4 Functions as a Pro‐Apoptotic Regulator in OCs

**FIGURE 3:**
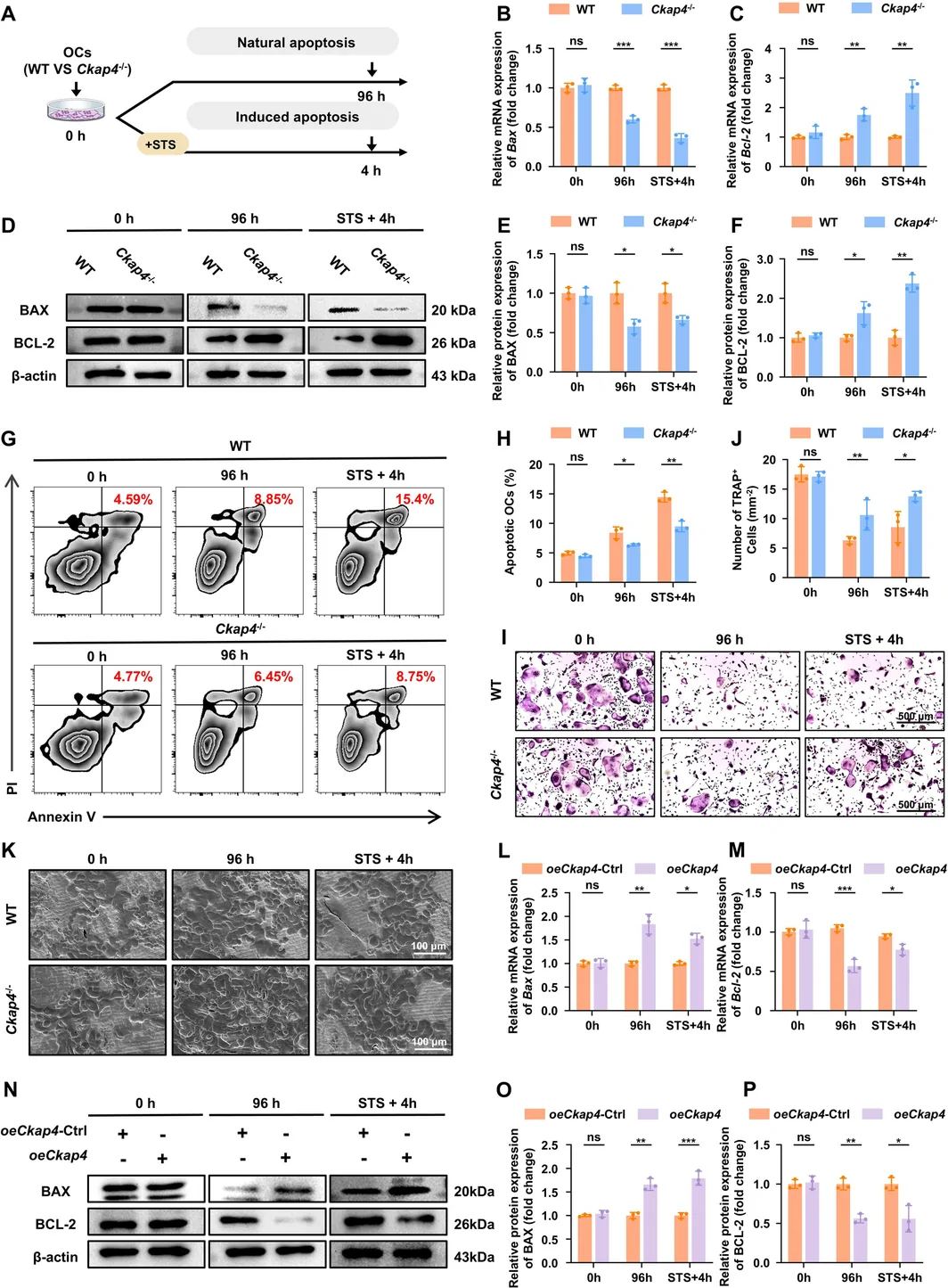
CKAP4 functions as a pro‐apoptotic regulator in OCs. (A) Experimental design for B‐P. OCs were compared between WT and *Ckap4* ^−/−^ groups for their natural apoptosis (over 96 h) and STS‐induced apoptosis (over 4 h). *Bax* (B) and *Bcl‐2* (C) mRNA expression in WT and *Ckap4* ^*−/−*^ OCs at 0, 96 and STS + 4 h. Expression was detected by RT‐qPCR, normalised to *β‐actin* and presented as fold change relative to the 0 h group (*n* = 3). Immunoblotting (D) and quantification of BAX (E) and BCL‐2 (F) protein levels in WT and *Ckap4* ^*−/−*^ OCs at 0, 96 and STS + 4 h. The expression level was normalised to β‐actin and the results are shown as fold change over the 0 h group (*n* = 3). Flow cytometry analysis (G) and quantification (H) of apoptotic OCs (Annexin V^+^/PI^+^) in WT and *Ckap4* ^*−/−*^ groups (*n* = 3). TRAP staining (I) and quantification of numbers of TRAP^+^ OCs per mm^2^ (J) in WT and *Ckap4* ^*−/−*^ OCs at 0 h, 96 h and STS + 4 (*n* = 3). Scale bar: 500 μm. (K) Scanning electron microscope (SEM) images of bone slices with WT and *Ckap4* ^*−/−*^ OCs at 0, 96 h and STS + 4 h. Scale bar: 100 μm. (L,M) *Bax* (L) and *Bcl‐2* (M) mRNA expression in *oeCkap4*‐Ctrl and *oeCkap4* OCs at 0 h, 96 h and STS + 4 h. Expression was detected by RT‐qPCR, normalised to *β‐actin* and presented as fold change relative to the 0 h group (*n* = 3). (N) The expression levels of BAX and BCL‐2 in OCs in *oeCkap4*‐Ctrl and *oeCkap4* OCs at 0, 96 h and STS + 4 were confirmed by WB assays. β‐actin was used as a loading control. (O, P) Quantification of BAX and BCL‐2 levels was normalised to β‐actin and the results are shown as fold change over the *oeCkap4*‐Ctrl group (*n* = 3). Results are shown as mean ± SD. Each dot indicates an individual sample. Student's *t*‐test (B–P) was performed. ***p < 0.001; **p < 0.01; *p < 0.05; ns, not significant.

To clarify the role of CKAP4 in regulating OC apoptosis, we generated global *Ckap4* knockout (*Ckap4* ^*−/−*^) mice and demonstrated the effectiveness of knockout by RT‐qPCR and WB assay (Figure S2A,B). Subsequently, we induced bone marrow cells from *Ckap4* ^*−/−*^ mice to harvest OCs and observed apoptosis processes of OCs under both natural condition (cultured for 96 h) and induced condition (treated with STS for 4 h) (Figure 3A). Notably, under both sets of conditions, *Ckap4* ^*−/−*^ OCs exhibited abnormal apoptotic phenotypes, characterised by attenuated upregulation of BAX and impaired downregulation of BCL‐2, suggesting that CKAP4 deficiency impairs the activation of the core apoptotic machinery (Figure 3B–F). Additionally, we performed flow cytometry gating OCs based on CTSK expression (Figure S2C) and detected their apoptosis rate (Figure 3G). Results demonstrated a significant reduction in the proportion of apoptotic OCs in the *Ckap4* ^*−/−*^ group relative to the wild‐type (WT) group (Figure 3G,H).TRAP staining of mature OCs revealed no significant difference in the number or area of TRAP‐positive (TRAP^+^) cells between the WT and *Ckap4* ^*−/−*^ groups at 0 h (Figures 3I,J and S2D), demonstrating that CKAP4 deficiency had no obvious effect on the maturation process of OCs. However, during the apoptotic process, the number and area of TRAP^+^ cells in the *Ckap4* ^*−/−*^ group were consistently higher than those in the WT group (Figures 3I,J and S2D), indicating that CKAP4 deficiency impairs the apoptotic sensitivity of OCs and thus increases the number of OCs.

Furthermore, to assess the impact of CKAP4 deficiency on OC bone resorption function, we induced the maturation of an equal number of WT and *Ckap4* ^−/−^ OCs on bone slices. After different treatments, the area of bone resorption lacunae on the surface of bone slices was detected. The results showed that there was no significant difference in the area of bone resorption lacunae between the two groups at 0 h (Figures 3K and S2E). This suggests that CKAP4 deficiency does not affect the bone resorption function of non‐apoptotic OCs. However, during both natural and induced apoptosis processes, the bone resorption area of *Ckap4* ^*−/−*^ OCs was significantly increased compared with the WT group, indicating that the bone destruction function of *Ckap4* ^*−/−*^ OCs was enhanced, which might be attributed to the reduced apoptosis of OCs caused by CKAP4 deficiency (Figures 3K and S2E). In addition, compared with the WT group, the expression levels of CTSK and TRAP, proteins related to bone resorption, were significantly increased in *Ckap4* ^*−/−*^ OCs, indicating that *Ckap4* ^*−/−*^ OCs exhibit less impairment of their bone resorption function during apoptosis (Figure S2F–H).

To further confirm the effect of CKAP on OC apoptosis, we overexpressed CKAP4 (*oeCkap4*) in OCs by LVs. Immunofluorescence showed nearly all OCs expressed GFP post‐treatment, indicating efficient lentiviral transduction (Figure S2I). RT‐qPCR and WB confirmed significantly increased CKAP4 expression at both mRNA and protein levels in the *oeCkap4* group compared to *oeCkap4*‐Ctrl (Figure S2J–K), verifying successful transfection of the CKAP4‐overexpressing LV. After that, we detected the expression of apoptosis‐related molecules. The results showed that at 0 h, there was no significant difference in the expression levels of BAX and BCL‐2 between the *oeCkap4*‐Ctrl and *oeCkap4* groups of OCs, indicating that CKAP4 overexpression had no effect on OC formation (Figure 3L–P). However, during natural or induced apoptosis, compared with the *oeCkap4*‐Ctrl group, the *oeCkap4* group of OCs showed a significant upregulation of BAX expression and a significant downregulation of BCL‐2 expression, indicating the role of CKAP4 overexpression in increasing the apoptotic susceptibility of OCs (Figure 3L–P). Consistently, TRAP staining revealed a significant reduction in the number and area of TRAP^+^ cells in the oeCkap4 group relative to the *oeCkap4*‐Ctrl group, confirming that CKAP4 overexpression accelerates OC apoptosis (Figure S2L–N). Taken together, the above experimental results in vitro confirmed that CKAP4 is related to the apoptosis susceptibility of OCs and CKAP4 deficiency hinders the apoptotic process of OCs.

### 3.4 Conditional *Ckap4* Knockout in OCs Aggravates Periodontitis‐Related Bone Resorption In Vivo

**FIGURE 4:**
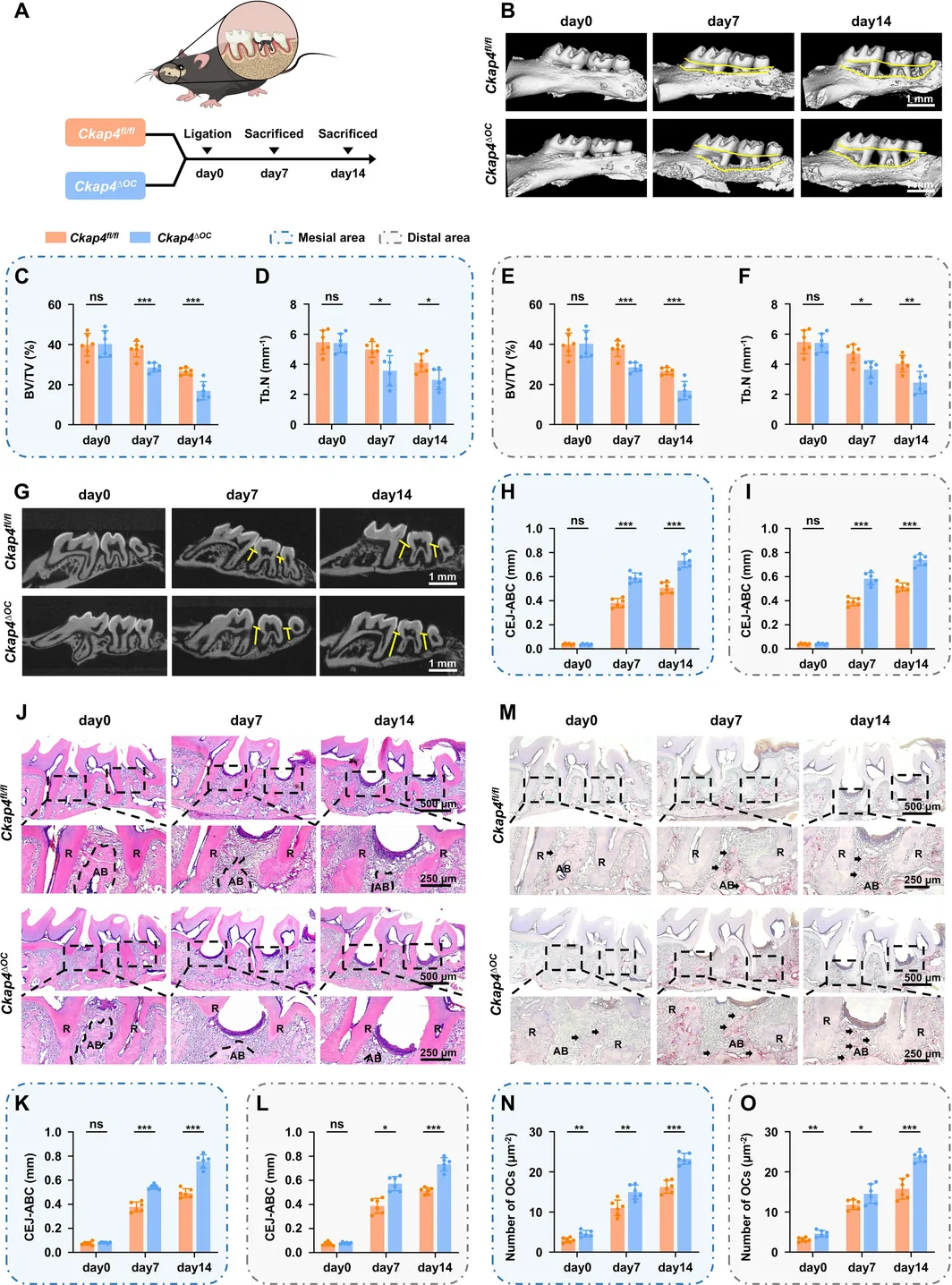
Conditional *Ckap4* knockout in OCs aggravates periodontitis‐related bone resorption in vivo. (A) Experimental design for B‐O. *Ckap4* ^*fl/fl*^ and *Ckap4* ^*ΔOC*^ mice were subjected to periodontitis induction through silk suture ligation, with tissue harvesting on Days 0, 7 and 14. (B) Micro‐CT three‐dimensional reconstructions of maxillae from *Ckap4* ^*fl/fl*^ and *Ckap4* ^*ΔOC*^ mice on Days 0, 7 and 14. Scale bar: 1 mm. Quantification of BV/TV (C, E) and Tb.N (D, F) in the mesial and distal areas of the second maxillary molar from *Ckap4* ^*fl/fl*^ and *Ckap4* ^*ΔOC*^ mice on Days 0, 7 and 14 (*n* = 6). Yellow lines indicate the distance of alveolar bone loss. Scale bar: 1 mm. (G) Micro‐CT sagittal sections of maxillae from *Ckap4* ^*fl/fl*^ and *Ckap4* ^*ΔOC*^ mice on Days 0, 7 and 14 (*n* = 6). Yellow lines indicate the distance of CEJ‐ABC. Scale bar: 1 mm. Quantification of CEJ‐ABC distance in the mesial (H) and distal (I) areas of the second maxillary molar from *Ckap4* ^*fl/fl*^ and *Ckap4* ^*ΔOC*^ mice on Days 0, 7 and 14 (*n* = 6). (J) H&E staining of maxillary molar sections from *Ckap4* ^*fl/fl*^ and *Ckap4* ^*ΔOC*^ mice (dashed boxes highlight periodontal tissue; R, root; AB, alveolar bone). Top panels, scale bar: 500 μm; bottom panels, scale bar: 250 μm. Quantification of CEJ‐ABC distance in the mesial (K) and distal (L) areas of the second maxillary molar based on J (*n* = 6). (M) TRAP staining of maxillary molar sections from *Ckap4* ^*fl/fl*^ and *Ckap4* ^*ΔOC*^ mice (dashed boxes highlight TRAP^+^ OCs). Top panels, scale bar: 500 μm; bottom panels, scale bar: 250 μm. Quantification of TRAP^+^ OC number in the mesial (N) and distal (O) areas of the second maxillary molar from *Ckap4* ^*fl/fl*^ and *Ckap4* ^*ΔOC*^ mice on Days 0, 7 and 14 (*n* = 6). Results are shown as mean ± SD. Each dot indicates an individual sample or mouse. Student's *t*‐test (C–O) was performed. ***p < 0.001; **p < 0.01; *p < 0.05; ns, not significant.

Next, we investigated the effect of CKAP4 on OC apoptosis in vivo. *Ctsk*‐iCre mice were crossed with *Ckap4* ^*flox/flox*^ (*Ckap4* ^*fl/fl*^) mice to generate *Ctsk*‐iCre::*Ckap4* ^*flox/flox*^ (*Ckap4* ^ΔOC^) mice, a conditional knockout model with *Ckap4* specifically deleted in OCs (Figure S3A) with efficient knockout validated by WB (Figure S3B). Experimental periodontitis was induced in *Ckap4* ^ΔOC^ and *Ckap4* ^*fl/fl*^ mice by silk ligation (Figure 4A). Micro‐CT (micro‐computed tomography) analysis showed no significant differences were observed in the alveolar bone between the two groups at Day 0, including the BV/TV ratio, the Tb.N (trabecular bone number) and the CEJ‐ABC distance on the mesial and distal sides of maxillary second molar (Figure 4B–L), indicating OC‐specific *Ckap4* knockout does not affect physiological alveolar bone remodelling. However, at Days 7 and 14 post‐ligation, *Ckap4* ^ΔOC^ mice exhibited significantly lower BV/TV and Tb.N, alongside greater CEJ‐ABC elevation, compared with *Ckap4* ^*fl/fl*^ controls (Figure 4B–I), with H&E staining validating this trend (Figure 4J–L), indicating that the destruction and resorption of alveolar bone in *Ckap4* ^ΔOC^ mice were more severe compared with *Ckap4* ^*fl/fl*^ mice. TRAP staining of periodontal tissues revealed significantly more OCs in *Ckap4* ^ΔOC^ mice than controls during periodontitis progression, particularly at days 7 and 14 post‐ligation (Figure 4M–O), which may be the reason for the more severe alveolar bone resorption in *Ckap4* ^ΔOC^ mice.

Since bone homeostasis depends on coupled OC and osteoblast activities, we also detected osteocalcin (OCN) in periodontal tissues. Immunofluorescence showed significantly lower OCN expression in *Ckap4* ^ΔOC^ mice than in controls at Days 7 and 14 post‐ligation (Figure S3C,D), indicating impaired periodontal osteogenesis in *Ckap4* ^ΔOC^ mice. This may stem from CKAP4 deficiency‐induced OC apoptosis impairment that disrupts OC‐osteoblast coupling [18]. These in vivo experimental periodontitis findings demonstrate that CKAP4 deficiency impairs OC apoptosis, leading to OC accumulation and subsequent exacerbation of alveolar bone resorption.

### 3.5 CKAP4 Activates the PERK‐CHOP Pathway to Mediate ERS‐Induced OC Apoptosis

**FIGURE 5:**
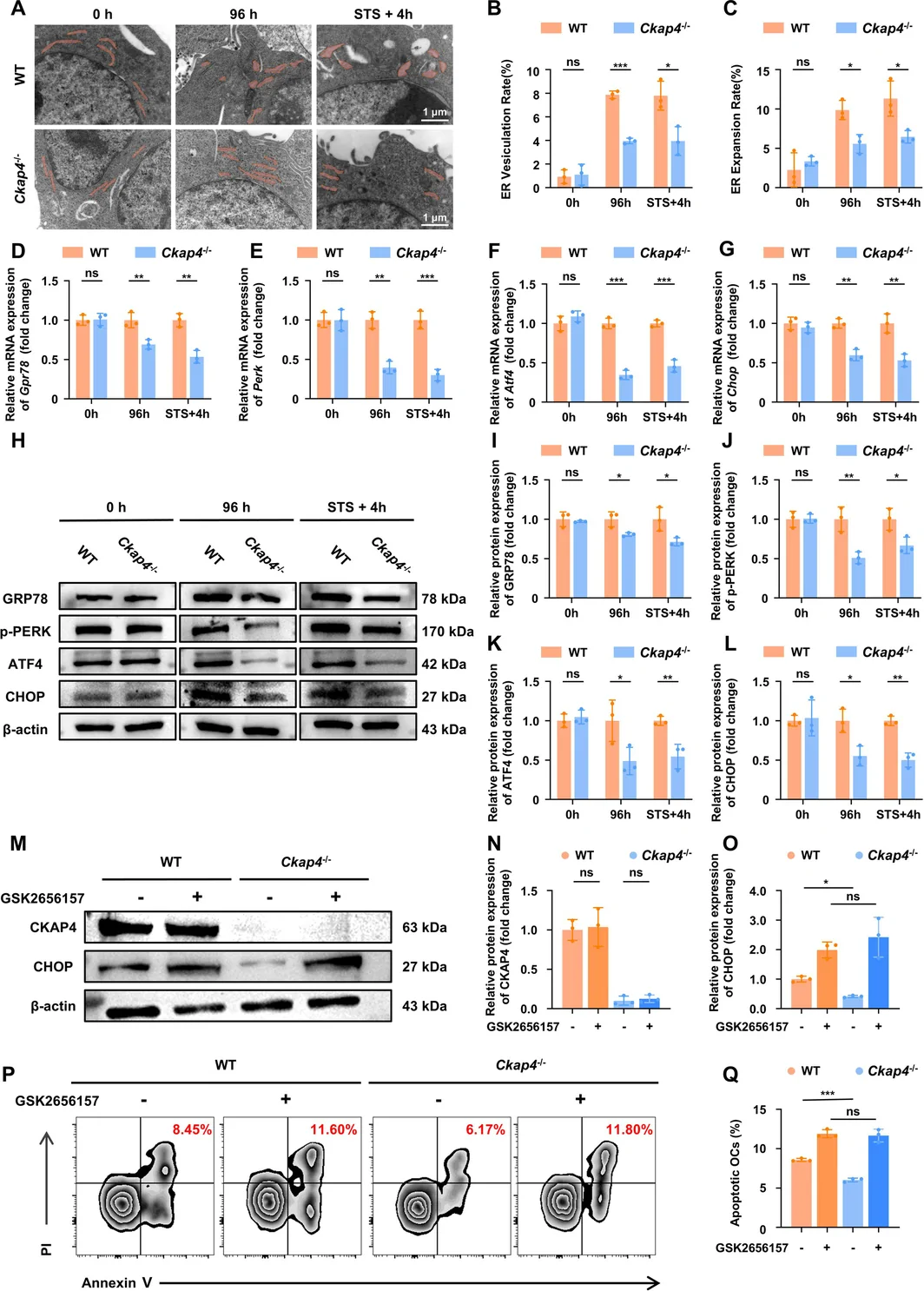
PERK‐CHOP pathway activation by CKAP4 drives ERS‐induced OC apoptosis. (A) TEM images of ER in WT and *Ckap4* ^*−/−*^ OCs at 0, 96 and STS + 4 h (red areas indicate ER). Scale bar: 1 μm. (B, C) Quantification of ER morphology‐related parameters: ER vesiculation rate and ER expansion rate (relevant to A) (*n* = 3). *Grp78* (D), *Perk* (E), *Atf4* (F) and *Chop* (G) mRNA expression in WT and *Ckap4* ^*−/−*^ OCs at indicated time points. Expression was detected by RT‐qPCR, normalised to *β‐actin* and presented as fold change relative to the 0 h group (*n* = 3). (H) Immunoblotting of GRP78, p‐PERK, ATF4, CHOP and β‐actin (loading control) in WT and *Ckap4* ^*−/−*^ OCs. (I‐L) Quantification of GRP78 (I), p‐PERK (J), ATF4 (K) and CHOP (L) protein levels from (H). The expression levels were normalised to β‐actin and the results are shown as fold change over the 0 h group (*n* = 3). (M) Immunoblotting of CKAP4, CHOP and β‐actin (loading control) in WT and *Ckap4* ^*−/−*^ OCs with/without GSK2656157 (PERK activator) treatment. Quantification of CKAP4 (N) and CHOP (O) protein levels in WT and *Ckap4* ^*−/−*^ OCs with/without GSK2656157 treatment from panel M. Expression was detected by WB assay, normalised to β‐actin and presented as fold change relative to the WT OCs without GSK2656157 treatment group (*n* = 3). Flow cytometry analysis (P) and quantification (Q) of apoptotic OCs (Annexin V^+^/PI^+^) in WT and *Ckap4* ^*−/−*^ OCs with/without GSK2656157 treatment (*n* = 3). Results are shown as mean ± SD. Each dot indicates an individual sample. Student's *t*‐test (B–L) and one‐way ANOVA with Tukey's post hoc test (N–Q) were performed. ***p < 0.001; **p < 0.01; *p < 0.05; ns, not significant.

Given that the subset of CKAP4, expressed on the cellular membrane, serves as Dickkopf‐1 (DKK1) receptor to activate the phosphoinositide 3‐kinase‐ protein kinase B (PI3K/AKT) pathway and promote cell survival and proliferation [19], we detected PI3K/AKT pathway‐related proteins in OCs. No expression differences were found between *Ckap4* ^*−/−*^ and WT OCs, indicating this pathway is dispensable for CKAP4‐mediated OC apoptosis (Figure S4A–C). We then focused on ERS, a CKAP4‐associated and apoptosis‐linked process [11, 20]. We analysed the ERS pathway of OCs with high and low *CKAP4* expression (*CKAP4* ^low^—OCs and *CKAP4* ^high^—OCs) in the aforementioned scRNA‐seq databases. The results showed that the ERS pathway was significantly downregulated in *CKAP4* ^low^—OCs (Figure S4D). To investigate whether *Ckap4* knockout would affect ERS level, we first observed the morphology of the ER by transmission electron microscopy (TEM). The results showed that at 0 h, the ER in both groups presented as flat cisternae with narrow lumens (Figure 5A–C). However, during natural and induced apoptosis, significant morphological changes in the ER of WT OCs were observed, including dilation and vesiculation, while such changes were not obvious in *Ckap4* ^−/−^ OCs, indicating an impaired ERS response in *Ckap4* ^−/−^ OCs (Figure 5A–C).

During ERS, glucose‐regulated protein 78 (GRP78) activates the unfolded protein response (UPR) pathway, which mainly consists of three signalling pathways: protein kinase RNA‐like endoplasmic reticulum kinase (PERK), inositol‐requiring enzyme 1 (IRE1) and activating transcription factor 6 (ATF6). The expression levels of these genes reflect ERS level [21]. Therefore, we detected the expression of these genes by qRT‐PCR. Results showed *Grp78* and *Perk* had comparable baseline expression in both groups at 0 h, but were significantly downregulated in *Ckap4* ^*−/−*^ OCs during apoptosis (vs. WT) (Figure 5D–L). No differences were observed for *Ire1α* or *Atf6* (Figure S4E,F). PERK is critical for ERS‐induced apoptosis, acting via its downstream target C/EBP homologous protein (CHOP) [21]. We then detected genes in the PERK pathway (*Grp78*, *Perk*, *Atf4*, *Chop*) and found their mRNA expression was significantly lower in *Ckap4* ^*−/−*^ OCs than in WT controls during apoptosis (Figure 5D–G), with WB confirming this trend (Figure 5H–L). The above results suggest that CKAP4 may regulate OC apoptosis induced by ERS through the PERK‐CHOP axis.

To validate this hypothesis, we treated OCs with GSK2656157 to promote PERK phosphorylation, thereby activating the PERK‐CHOP pathway. GSK2656157 significantly upregulated CHOP to comparable levels in WT and *Ckap4* ^*−/−*^ OCs (Figure 5M–O). Notably, GSK2656157 treatment restored the apoptotic rate of *Ckap4* ^*−/−*^ OCs to WT levels (Figure 5P,Q), confirming the PERK‐CHOP pathway is key for CKAP4‐mediated ERS. We also treated OCs with GSK2606414 (to inhibit PERK phosphorylation), which reduced CHOP to comparable baseline levels in both groups (Figure S4G–I). Whether PERK is activated or inhibited, there is no difference in the expression of CKAP4 in OCs of the WT group (Figures 5P,Q and S4G–I). Consistently, flow cytometry showed GSK2606414 reduced apoptotic rates to similar levels in WT and *Ckap4* ^*−/−*^ OCs (Figure S4J–K). The above results demonstrate that CKAP4 deficiency attenuates ERS by suppressing the PERK‐CHOP pathway, thereby inhibiting OC apoptosis.

### 3.6 CKAP4 Promotes FOXO3 Expression and Activates the PERK‐CHOP Pathway

**FIGURE 6:**
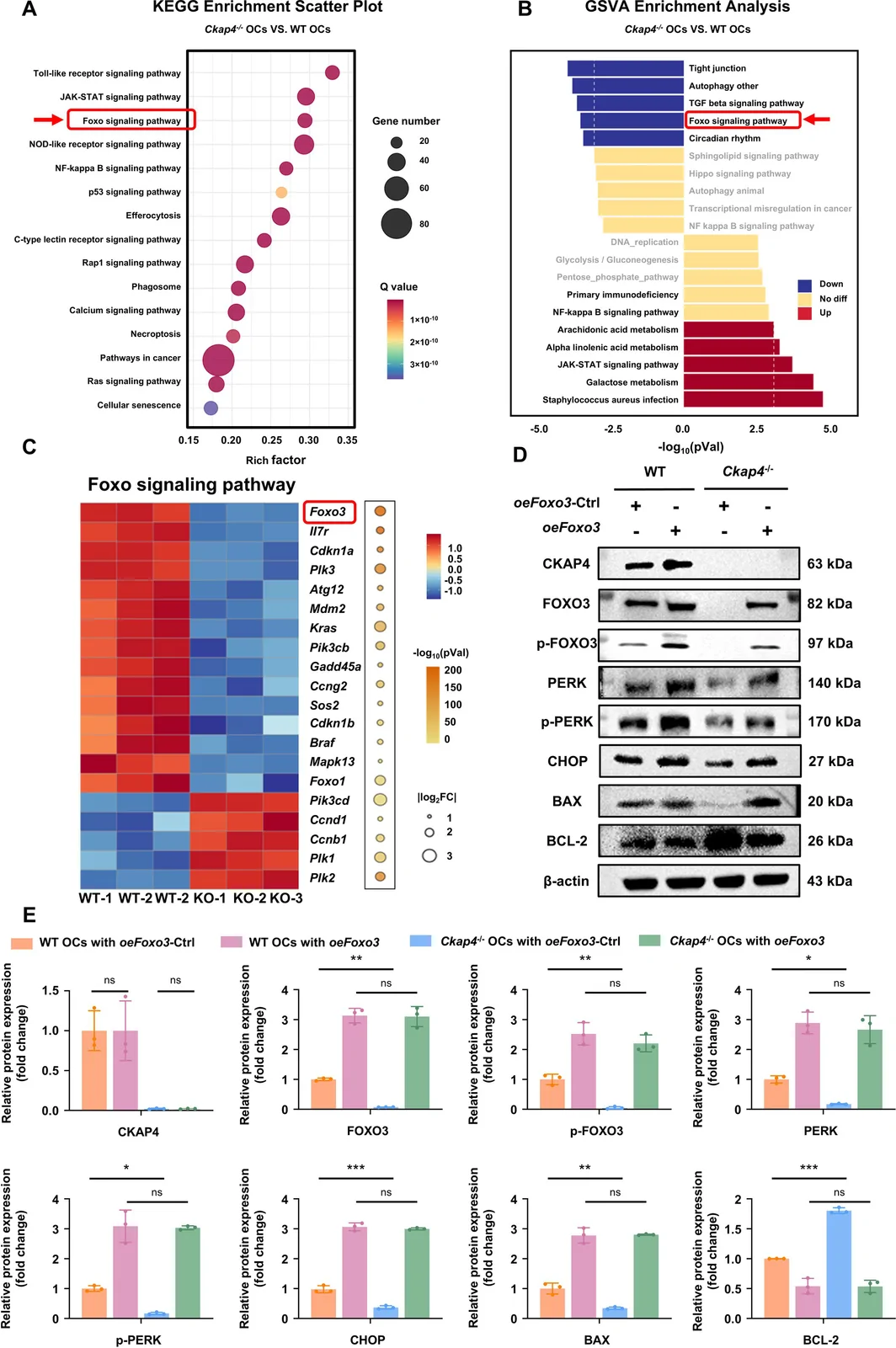
CKAP4 promotes FOXO3 expression and activates the PERK‐CHOP pathway. (A) KEGG enrichment scatter plot comparing *Ckap4* ^*−/−*^ OCs and WT OCs, with the *Foxo* signalling pathway highlighted (red arrow). (B) GSVA enrichment analysis comparing *Ckap4* ^*−/−*^ OCs and WT OCs, with the *Foxo* signalling pathway highlighted (bar colour indicates pathway regulation direction: red for upregulation, yellow for no difference, blue for downregulation) (C) Clustered heatmap of gene expression in the *Foxo* signalling pathway with *Foxo3* highlighted (*n* = 3). (D) Immunoblotting of CKAP4, FOXO3, p‐FOXO3, PERK, p‐PERK, CHOP, BAX, BCL‐2 and β‐actin (loading control) in WT and *Ckap4* ^−/−^ OCs with LV‐*oeFoxo3* or LV‐*oeFoxo3*‐Ctrl treatment. (E) Quantification of protein levels from (D). Expression was detected by WB assay, normalised to β‐actin and presented as fold change relative to WT OCs with LV‐*oeFoxo3*‐Ctrl group (*n* = 3). Results are shown as mean ± SD. Each dot indicates an individual sample. One‐way ANOVA with Tukey's post hoc test (E) were performed. ***p < 0.001; **p < 0.01; *p < 0.05; ns, not significant.

To elucidate how CKAP4 regulates the PERK‐CHOP pathway, we performed RNA sequencing on *Ckap4* ^*−/−*^ and WT OCs after 96 h of culture (natural apoptosis), identifying 1325 upregulated and 1308 downregulated genes in *Ckap4* ^*−/−*^ cells (Figure S5A). GSEA showed that apoptosis and UPR pathways were significantly downregulated in *Ckap4* ^*−/−*^ OCs (Figure S5B), corroborating our prior results. Subsequent KEGG analysis revealed DEG enrichment in apoptosis‐related pathways, such as JAK–STAT, *Foxo* (forkhead box O), Rap1 and p53 (Figure 6A). Notably, only the *Foxo* gene set was significantly downregulated in *Ckap4* ^*−/−*^ OCs (Figures 6B and S5C,D). This downregulation aligns with PERK‐CHOP pathway suppression in *Ckap4* ^*−/−*^ OCs, implying that the *Foxo* family may play a critical role in mediating CKAP4's regulation of the PERK‐CHOP pathway. Furthermore, we found among the *Foxo* family, transcription factor *Foxo3* exhibited the most significant downregulation in *Ckap4* ^*−/−*^ OCs (Figure 6C). RT‐qPCR validation of four major *Foxo* family transcription factors (*Foxo1*, *Foxo3*, *Foxo4*, *Foxo6*) [22] confirmed this trend (Figure S5D). Consistent with sequencing data, *Foxo3* was significantly downregulated in *Ckap4* ^*−/−*^ OCs (Figure S5D). Interestingly, WB results showed that FOXO3 was barely expressed in *Ckap4* ^*−/−*^ OCs (Figure S5E). As FOXO3 promotes *Perk* transcription in cancer cells [22], we hypothesise that FOXO3 is the key factor through which CKAP4 regulates the PERK‐CHOP pathway.

To validate this, we generated *Foxo3*‐knockdown (*shFoxo3*) and ‐overexpressing (*oeFoxo3*) OCs via lentiviral transduction, with empty vector‐infected OCs as controls (*shFoxo3*‐Ctrl/*oeFoxo3*‐Ctrl) (Figure S5F,G). WB confirmed efficient FOXO3 modulation (Figure S5H–K). We then assessed PERK‐CHOP axis protein levels after 96 h of post‐maturation culture (natural apoptosis) (Figure 6D,E, S5L,M). *Foxo3* overexpression reversed PERK‐CHOP pathway inhibition in CKAP4‐deficient OCs and upregulated apoptosis‐related proteins (Figure 6D,E). Conversely, *Foxo3* knockdown in WT OCs significantly suppressed the PERK‐CHOP axis and cell apoptosis (Figure S5L,M). Notably, CKAP4 expression remained unchanged in both manipulation groups (Figures 6D,E and S5L,M). However, co‐immunoprecipitation (Co‐IP) showed no direct interaction between CKAP4 and FOXO3 (Figure S5N), suggesting that CKAP4 may regulate the expression of FOXO3 through an indirect mechanism.

These results confirm that CKAP4 promotes the PERK‐CHOP pathway via FOXO3, thereby regulating ERS‐induced OC apoptosis.

## 4 Discussion

**FIGURE 7:**
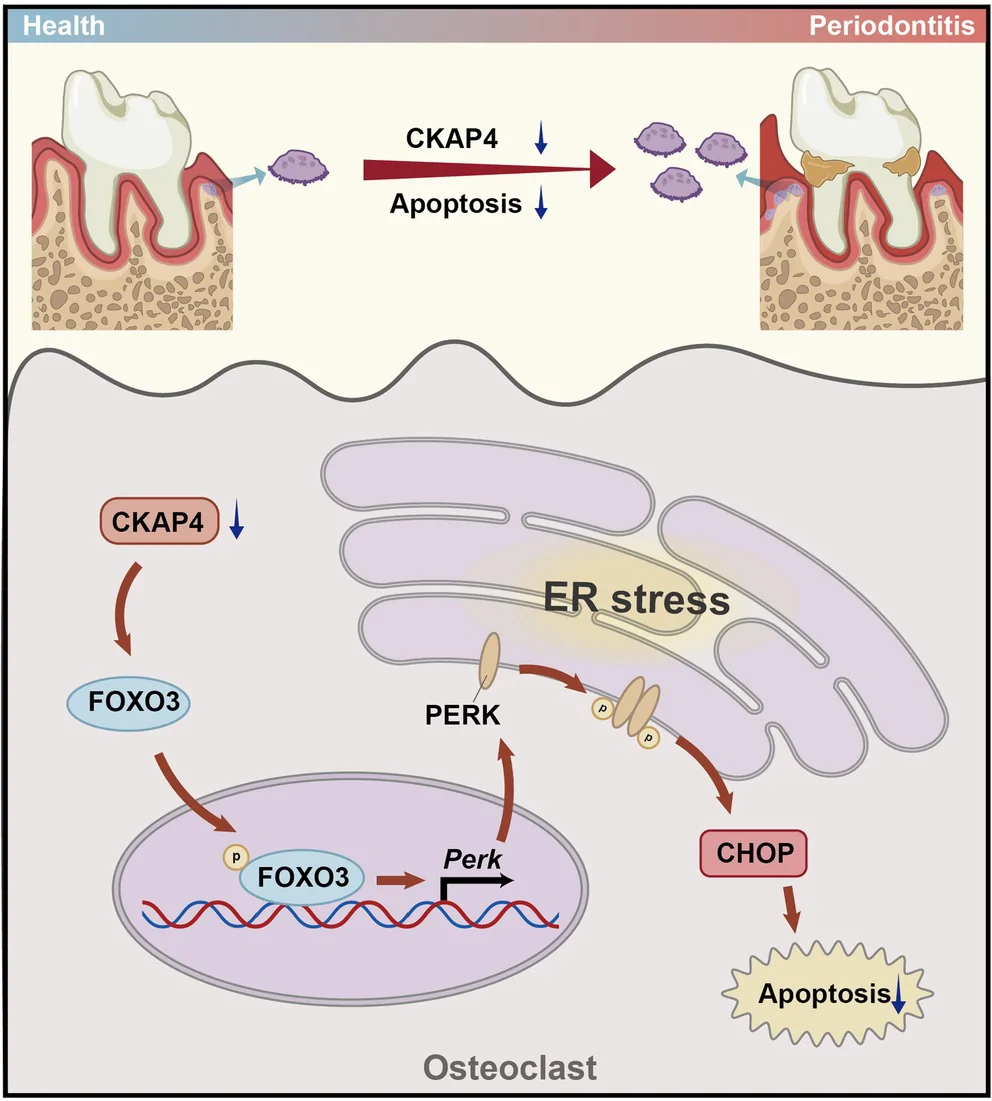
CKAP4 Regulates ERS‐induced Apoptosis in OCs through FOXO3 in periodontitis. CKAP4 promotes the transcription of PERK via FOXO3 in OCs, thereby facilitating ERS‐induced apoptosis. In periodontitis, the reduction of CKAP4 in OCs leads to decreased OC apoptosis, which exacerbates alveolar bone resorption.

OCs are the key effector cells during alveolar bone resorption in periodontitis, yet changes in their apoptotic capacity during periodontitis and the underlying mechanisms have rarely been reported [15]. In this study, we found that the apoptotic capacity of OCs is significantly downregulated in periodontitis. We also unveiled an unrecognized mechanism that CKAP4 can regulate FOXO3 and mediate OC apoptosis induced by ERS through the PERK‐CHOP pathway (Figure 7). This finding provides CKAP4 as a new molecular target for alleviating alveolar bone resorption in periodontitis.

Our study identified the downregulation of the OC apoptosis pathway in periodontitis through single‐cell RNA sequencing analysis of human periodontal tissues and further validated this finding by a mouse animal model. Currently, research on bone resorption in periodontitis primarily focuses on the functions of immune cells and osteoblasts, as well as their regulatory effects on OC differentiation, and such studies often treat OCs merely as a ‘marker’ of bone resorption capacity [23]. However, as the primary effector cells responsible for bone resorption. The changes in OC apoptotic capacity and the underlying mechanisms during periodontitis remain rarely elucidated. In osteoarthritis, another inflammatory disease characterised by prominent bone resorption, a reduction in OC apoptotic capacity has been observed, yet no further validation or mechanistic exploration of this phenomenon has been conducted [9]. Researches on OC apoptosis predominantly centred on osteoporosis. Beyond the classical apoptotic pathways, the RANKL/OPG pathway and NF‐κB pathway, which are both closely associated with OC differentiation, have been shown to bidirectionally regulate OC survival [4]. In our study, the initial number of OCs did not change before and after *Ckap4* knockout. Additionally, mice with OC‐specific *Ckap4* knockout exhibited no significant alterations in alveolar bone mass under normal (non‐inflammatory) conditions. These results suggest that CKAP4‐mediated regulation of OC apoptosis in periodontitis is independent of OC differentiation pathways and tightly linked to the inflammatory response.

CKAP4 has been reported as a key transmembrane protein that anchors ER membranes to the microtubule cytoskeleton, preventing random ER expansion and maintaining the integrity of ER tubular and sheet domains [20]. Interestingly, our study found that after *Ckap4* knockout, the structure of the ER becomes more stable than that in WT OCs when ERS occurs, with fewer signs of swelling or vesiculation. This result suggests that the function of CKAP4 in regulating ER structure is not static but dynamically modulated by the cellular stress state. Previous studies highlighting CKAP4's ER‐stabilising role were mostly conducted under basal (non‐stress) conditions in tumour cells [20]. However, our data indicate that when ERS is triggered (e.g., by inflammatory cytokines or unfolded protein accumulation, which are prevalent in periodontitis [24]), CKAP4 may undergo a functional shift: instead of serving as a structural scaffold, it may prioritise participation in ERS signalling cascades. As supported by a recent study, CKAP4 can assist the ER‐phagy protein FK506‐binding protein‐like (FKBPL) in mediating ER fragmentation during ERS. In summary, our findings support that the function of CKAP4 in regulating ER structure may be dependent on the cellular stress state and that CKAP4 can undergo a dynamic shift from maintaining ER structure to participating in ERS signalling.

OC‐osteoblast coupling plays a crucial role in maintaining bone homeostasis. In our in vivo experiments, we found that the expression of osteoblast‐related protein OCN in periodontal tissues was significantly reduced after specific knockout of CKAP4 in OCs. This suggests that the regulation of OCs by CKAP4 may affect OC‐osteoblast coupling. According to reports, apoptotic bodies formed during OC apoptosis can transfer pro‐osteogenic signals (e.g., Sphk1, TGF‐β1) to adjacent osteoblasts, thereby promoting osteoblast differentiation and mineralisation [18]. In our in vitro assays, we further observed that CKAP4 deficiency significantly inhibited OC apoptosis. Therefore, we speculate that the reduction in OCN may be attributed to the inhibition of OC apoptosis and the subsequent decrease in apoptotic bodies caused by CKAP4 deficiency. This indicates that CKAP4 may be a key regulatory molecule for maintaining bone homeostasis via modulating OC apoptosis and subsequent OC‐osteoblast coupling. However, the limitation of our work is lack of direct evidence for the altered production of OC‐derived apoptotic bodies and the expression of key coupling molecules in the *Ckap4* knockout model, which requires exploration in our future work.

Through transcriptome sequencing analysis, we identified the *Foxo* pathway that was significantly downregulated after *Ckap4* knockout. Furthermore, we verified that FOXO3 mediates the regulation of CKAP4 on OC apoptosis by performing *Foxo3* knockdown and overexpression experiments. Notably, FOXO3 and PERK engage in complex regulatory crosstalk [25]. PERK can activate FOXO3 through various mechanisms [26]. Conversely, FOXO3 can feedback to regulate PERK activity by binding to the PERK promoter and enhancing its transcription [27]. This bidirectional regulation may allow for fine‐tuning of the apoptotic response to the periodontal inflammatory microenvironment. CKAP4 may be involved in this bidirectional regulation, thus contributing to the aforementioned functional switch of cells under ERS conditions.

Notably, the PI3K/AKT pathway, a major mediator of FOXO3 degradation [28], remained unchanged upon *Ckap4* knockout, whereas FOXO3 transcription was significantly reduced. This imbalance between decreased synthesis and unaltered degradation likely explains the nearly undetectable FOXO3 expression. Although phosphorylated CKAP4 can enter the nucleus to regulate gene expression [29], our co‐IP results exclude direct CKAP4‐FOXO3 binding, suggesting indirect regulation. This represents an important unresolved issue that warrants further investigation.

Accumulating evidence indicates that FOXO3 and the PERK‐CHOP axis play important roles in periodontitis progression. FOXO3 serves as a key positive regulator of periodontal homeostasis [30, 31]. Our previous study demonstrated that regulation of PERK activity significantly influences alveolar bone resorption in periodontitis [32]. Collectively, FOXO3 and the PERK‐CHOP axis are critical for periodontal bone homeostasis. However, the in vivo mechanisms of the FOXO3/PERK‐CHOP axis require further validation.

In conclusion, our findings underscored that the apoptotic capacity of OCs in periodontitis is significantly downregulated, which further explains the irreversible alveolar bone resorption observed in periodontitis. Moreover, our results disclosed that CKAP4 can mediate ERS via FOXO3 to promote OC apoptosis, while the expression of CKAP4 is significantly downregulated in OCs during periodontitis. This mechanism provides a novel molecular target for inhibiting alveolar bone resorption in periodontitis.

A key limitation of our study is the lack of elucidation regarding the upstream regulatory mechanisms responsible for CKAP4 downregulation in periodontal OCs. Our findings clearly demonstrate that reduced CKAP4 expression impairs OC apoptosis and exacerbates alveolar bone loss. However, the factors driving CKAP4 downregulation remain unknown. Potential mechanisms may include transcriptional repression by dysregulated transcription factors, post‐transcriptional regulation by microRNAs or long non‐coding RNAs [33] or post‐translational modifications (e.g., ubiquitination‐mediated degradation [34]) induced by the inflammatory periodontal microenvironment. Future investigations are warranted to identify these upstream regulators and to further explore the role of CKAP4 in OC‐osteoblast coupling, as this knowledge will enhance our understanding of the molecular cascades governing periodontal bone loss and provide more precise therapeutic targets for restoring CKAP4 function.

## Author Contributions

B.L. and C.S. conceived the study; J.C., D.T. and W.W. contributed to conception, study design, data collection and interpretation, drafted and revised the manuscript; Huiyu Wang, X.H, J.Y. and Y.W. conducted data analysis; J.G, Hanzhe Wang, W.L. and Y.L. provided technical assistance for data analysis. All authors gave their final approval and agreed to be responsible for this paper.

## Funding

This work was supported by the National Key Research and Development Program of China (2021YFA1100600), the National Natural Science Foundation of China (82370927), Science and Technology Program of Xi'an (25ZQRC00027) and the Shaanxi Provincial Key Research and Development Program (2023‐ZDLSF‐49 and 2024SF‐GJHX‐29).

## Ethics Statement

This study was carried out in compliance with the Declaration of Helsinki and all experiments followed Fourth Military Medical University Institutional Animal Care and Use Committee (IACUC) guidelines (IACUC‐20241268).

## Conflicts of Interest

The authors declare no conflicts of interest.

## Supporting information


**Data S1:** cpr70208‐sup‐0001‐supinfo.docx.
**Figure S1:** Single‐cell transcriptomic analyses of periodontal cells.
**Figure S2:** Verification and functional detection of *Ckap4* knockout and overexpression in OCs.
**Figure S3:** Generation of OC‐conditional *Ckap4* knockout mice and OCN expression analysis.
**Figure S4:** PI3K‐AKT pathway is not involved in CKAP4‐mediated OC apoptosis and PERK is involved in the regulation of CHOP by CKAP4.
**Figure S5:** Transcriptomic analysis and FOXO3 viral manipulation validation.
**Figure S6:** Uncropped pictures of the western blot bands presented in this study.
**Figure S7:** Uncropped pictures of the western blot bands presented in this study.
**Table S1:** Oligonucleotides used for genotyping.
**Table S2:** Antibodies used for WB.
**Table S3:** Oligonucleotides used for RT‐qPCR.
**Table S4:** Oligonucleotides of LV.

## Data Availability

The authors confirm that data supporting the study's findings are available in the paper and its Supporting Information. The RNA‐seq data generated in this study are available in the GEO under the accession number PRJNA1394630.
