# Supplementary material for: CKAP4 Regulates ERS‐Induced Apoptosis in Osteoclasts Through FOXO3 in Periodontitis
Source: Cell Prolif. 2026 Apr 13:e70208. Online ahead of print. doi: 10.1111/cpr.70208 (PMC13325814; doi:10.1111/cpr.70208)
Supplement: Supplementary file 1 — Data S1: cpr70208‐sup‐0001‐supinfo.docx. Figure S1: Single‐cell transcriptomic analyses of periodontal cells. Figure S2: Verification and functional detection of Ckap4 knockout and overexpression in OCs. Figure S3: Generation of OC‐conditional Ckap4 knockout mice and OCN expression analysis. Figure S4: PI3K‐AKT pathway is not involved in CKAP4‐mediated OC apoptosis and PERK is involved in the regulation of CHOP by CKAP4. Figure S5: Transcriptomic analysis and FOXO3 viral manipulation validation. Figure S6: Uncropped pictures of the western blot bands presented in this study. Figure S7: Uncropped pictures of the western blot bands presented in this study. Table S1: Oligonucleotides used for genotyping. Table S2: Antibodies used for WB. Table S3: Oligonucleotides used for RT‐qPCR. Table S4: Oligonucleotides of LV. [file CPR-9999-e70208-s001.docx]

**Supplementary Materials**

CKAP4 Regulates ER Stress-induced Apoptosis in Osteoclasts through FOXO3 in Periodontitis

Jiayu Cheng^1^, Dihao Tao^1^, Wenzhe Wang^1^, HuiYu Wang^1^, Hanzhe Wang^1^, Yufan Liang^1^, Jia Guo^1^, Wenhao Liu^1^, Yiming Wang^1^, Jianhua Yang^1^, Xiaoning He^1^, Ce Shan^1^, and Bei Li^1^

^1^ State Key Laboratory of Oral & Maxillofacial Reconstruction and Regeneration, National Clinical Research Center for Oral Diseases. Shaanxi International Joint Research Center for Oral Diseases, Center for Tissue Engineering, School of Stomatology, The Fourth Military Medical University, Xi'an, China.

Correspondence: Bei Li([libei2021@fmmu.edu.cn](mailto:libei2021@fmmu.edu.cn)) and Ce Shan (ce_shan2024@163.com)

These authors contributed equally: Jiayu Cheng, Dihao Tao, Wenzhe Wang

**Supplementary methods**

**TRAP Staining**

For cellular samples, TRAP staining was conducted using a TRAP Staining Kit (AMK1002-010; Amizona Scientific LLC, China) in accordance with the manufacturer’s protocol. Briefly, cells were fixed with 4% paraformaldehyde (PFA) for 20 minutes, followed by incubation with TRAP staining solution at 37 °C for 1 hour.For sections， deparaffinize paraffin sections in xylene (I, II) (10 min each), rehydrate via graded ethanol (100% to 70%, 5 min/step), and rinse with distilled water (5 min). Then incubate sections in pre-warmed TRAP staining solution (with naphthol AS-BI phosphate, tartrate-resistant buffer, fast red violet LB salt) at 37°C. Terminate with water, counterstain with hematoxylin (30s-1min), differentiate (1% HCl-ethanol, 5-10s), blue (5 min).

OCs were defined as TRAP-positive cells with three or more nuclei. Images were acquired via an optical microscope, and quantity and area of OCs were measured with ImageJ software.

**Flow cytometry**

Treated OCs were harvested and washed once with PBS. For intracellular CKAP4 staining, cells were fixed by adding 200 µL fixation working solution per sample for 60 min at room temperature. Subsequently, 100 µL permeabilization buffer supplemented with anti-CKAP4 antibodies (ab302539, 1:1000 dilution; Abcam, UK) was added, and the mixture was incubated on ice for 30 min. Following three washes, cells were stained with fluorophore-conjugated secondary antibodies for 30 min on ice. OC apoptosis was detected using the Annexin V-FITC/PI Apoptosis Detection Kit (BD Biosciences, USA). All data were collected using an LSR Fortessa flow cytometer (BD Biosciences, USA) and analyzed via FlowJo 10.0 software.

**Bone resorption assay**

The bone resorption assay was performed using sterilized cortical bovine bone slices (Amizona Scientific LLC, China). An equal number of BMDMs were seeded onto bone slices in osteoclastogenic mediu. Following culture or treatment, the bone slices were fixed with 4% paraformaldehyde. Subsequently, the cells were gently scraped off the bone slices using a soft brush. A thin gold film was then sputtered onto the dry bone slices to ensure electrical conductivity. The morphology of the surface bone resorption pit was subsequently observed and imaged using a scanning electron microscope (SEM; EIKO Engineering, Japan).

**Histology staining**

Fresh samples were fixed in 4% PFA at 4°C overnight, with subsequent decalcification in 17% EDTA (pH 7.4) for 21 days. The samples were then dehydrated using a graded ethanol series and embedded in paraffin. Vertical sections of 4 μm thickness were obtained from the tissues. The sections were stained with H&E and TRAP kits as per the manufacturers’ instructions, and images were captured via an optical microscope. The CEJ-ABC distance and the number of TRAP-positive cells (OC markers), were quantified via ImageJ software.

**Transmission electron microscopy (TEM)**

First, OCs were digested with trypsin, washed with PBS, and fixed for 2 h using freshly prepared fixative. Next, the fixed cells were incubated with 1% osmium tetroxide for 2 h, dehydrated through a graded ethanol series, and embedded in epoxy resin. Ultrathin sections were prepared with a Leica EM UC7 (Leica, Germany) and stained with uranyl acetate and lead citrate. Finally, the cells were analyzed by a transmission electron microscope (Hitachi, Japan).

**Immunofluorescence**

For cellular immunofluorescence, BMDMs were induced to differentiate into OCs in confocal-compatible microplates, followed by 0, 48, 96 h of culture or staurosporine (STS; Cell Signaling Technology, USA) treatment to induce apoptosis. After fixation, cells were blocked with goat serum (Sigma-Aldrich, USA) and permeabilized with 0.3% Triton X-100 (Sigma-Aldrich, USA), then incubated with anti-CKAP4 antibody (ab302539, 1:200; Abcam, UK) at 4 °C overnight. Fluorophore-conjugated secondary antibodies (1:500; Invitrogen, USA) were added and incubated at 37 °C for 2 h in the dark, followed by DAPI counterstaining for 15 min. For tissue sections, deparaffinization, antigen retrieval, and antibody incubation steps were performed as described in the Supplementary Methods. Primary antibodies included anti-CKAP4 (ab302539, 1:1000; Abcam, UK), anti-CTSK (TA375221, 1:200; OriGene, USA), and anti-Osteocalcin (ab93876, 1:200; Abcam, UK). TUNEL assay was conducted using the TUNEL Apoptosis Assay Kit (Servicebio, China) to detect OC apoptosis. Fluorescent images were acquired with a confocal scanning laser microscope (FV3000, Olympus, Japan) and analyzed by ImageJ software.

**
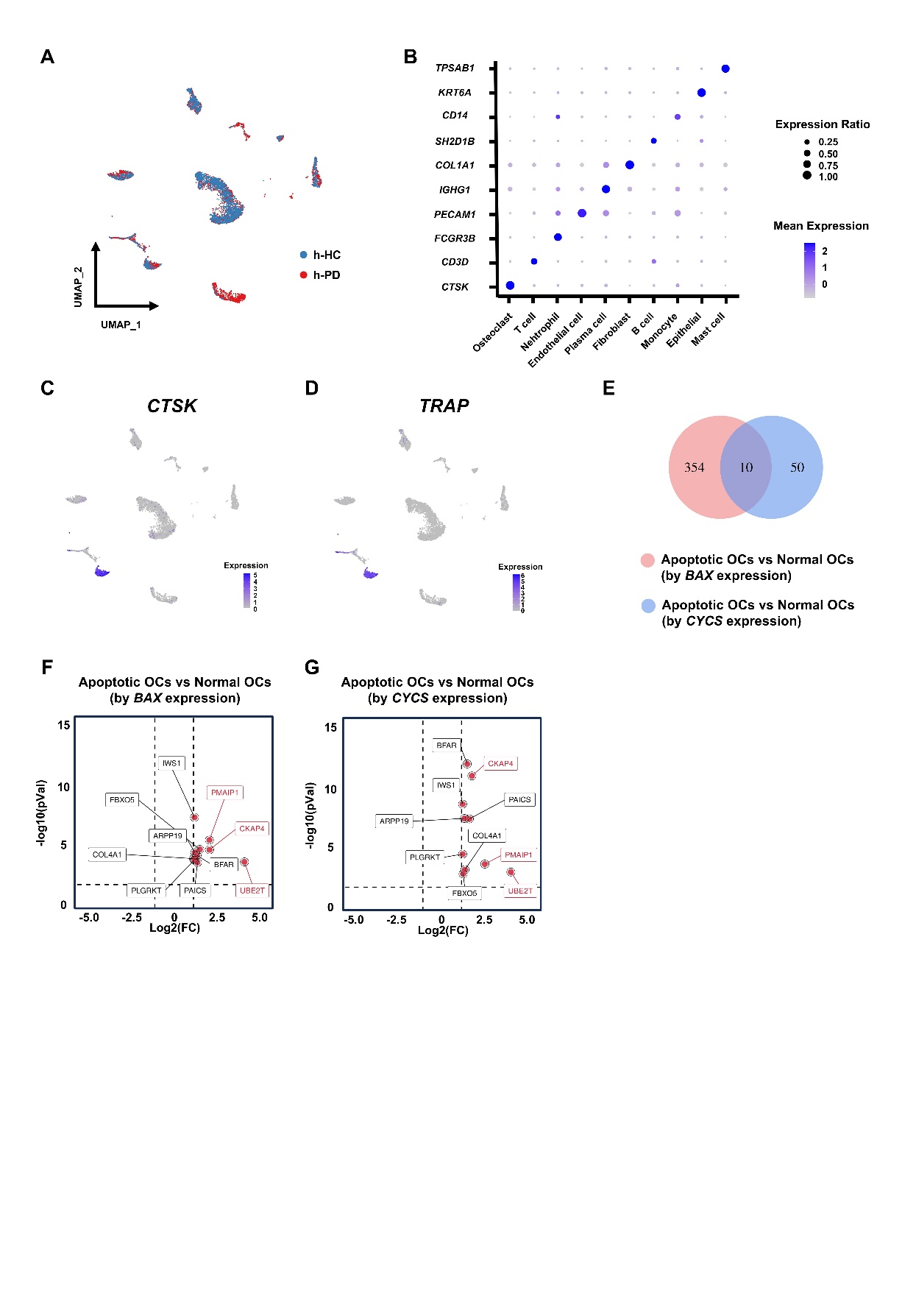
Figure S1 | Single-cell transcriptomic analyses of periodontal cells.**

(A) UMAP visualization of single cells from h-HC (blue) and h-PD (red) samples, showing cellular distribution across groups. (B) Dot plot illustrating expression patterns of marker genes across different cell types (x-axis). Dot size represents expression ratio, and color intensity represents mean expression level. (C–D) UMAP feature plots showing expression distribution of *CTSK* (C) and *ACP5* (D) across cells, with color scale indicating gene expression level. (E) Venn diagram depicting overlap of DEGs between apoptotic OCs vs normal OCs by *BAX* expression (pink) and *CYCS* expression (blue). (F–G) Volcano plot of DEGs between apoptotic OCs vs normal OCs by *BAX* expression (F) and *CYCS* expression (G). Red dots represent the above ten identical DEGs in e. The red gene name labels indicate the three genes with greatest differences in both figures.

Results are shown as mean ± SD. Each dot indicates an individual sample. ****P* < 0.001; ***P* < 0.01; **P* < 0.05; ns, not significant.


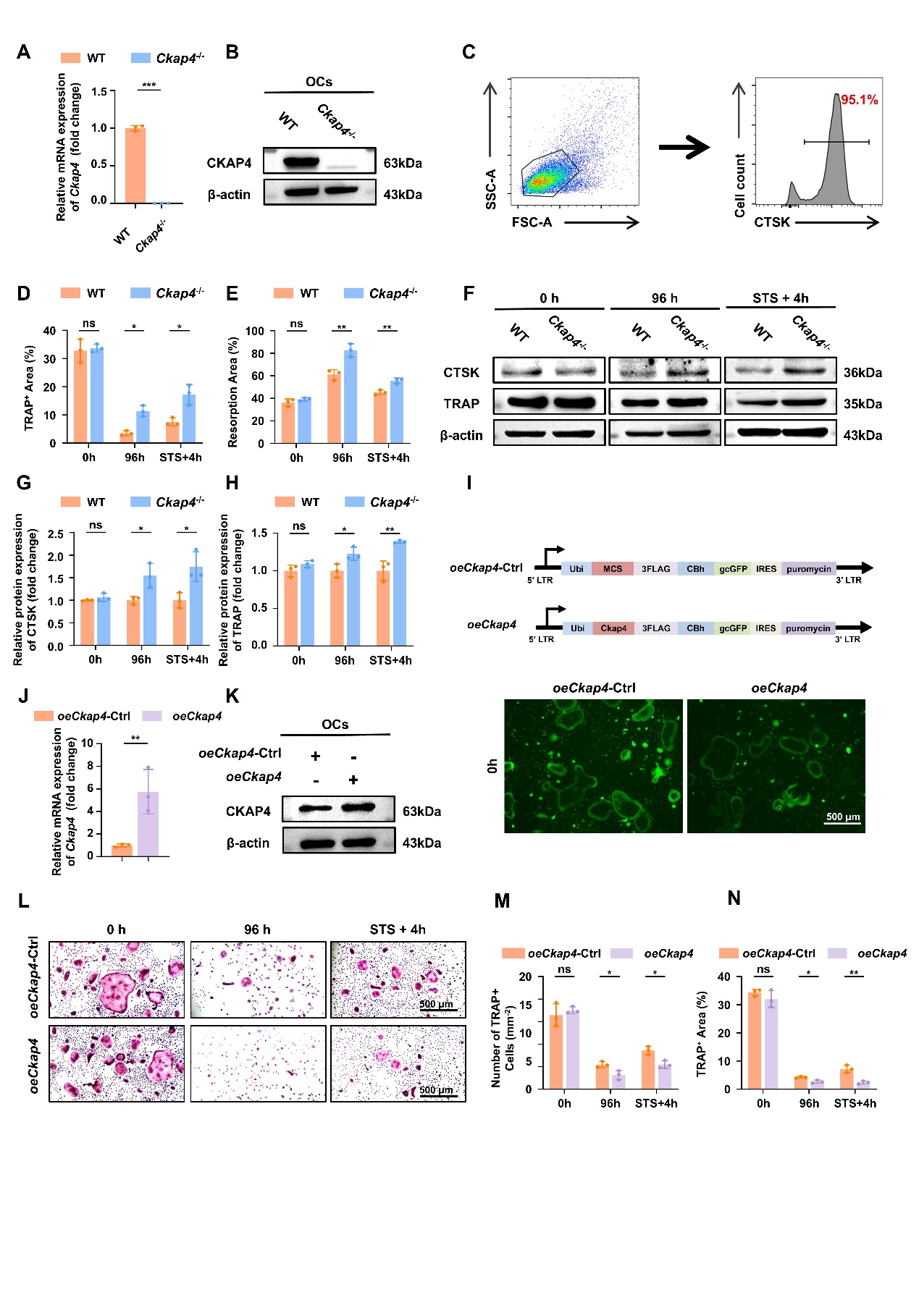


**Figure S2 | Verification and functional detection of *Ckap4* knockout and overexpression in OCs.**

(A) *Ckap4* mRNA expression in WT and *Ckap4^-/-^* OCs, measured by qRT-PCR shown as fold change over WT group (*n* = 3). (B) Immunoblotting of CKAP4 protein levels in WT and *Ckap4^-/-^* OCs, normalized to *β-actin*, and shown as fold change over WT group (*n* = 3). (C) Gating strategy for OCs (SSC-A⁺/FSC-A⁺) and flow cytometry analysis of CTSK expression in OCs. (D) TRAP⁺ area percentage in WT and *Ckap4^-/-^* OCs under different conditions from figure 3I (*n* = 3). (E) Resorption area percentage of WT and *Ckap4^-/-^* OCs on bone slices at indicated time points from figure 3k (*n* = 3). (F–H) Immunoblotting (h) and quantification of CTSK (F) and TRAP (H) protein levels in WT and *Ckap4^-/-^* OCs shown as fold change over 0h group (*n* = 3). (I) Microscopy images of OCs transfected with LV-Ctrl or LV-*Ckap4* (both expressing GFP). (J) *Ckap4* mRNA expression in LV-oe*Ckap4-*Ctrl- and LV-oe*Ckap4*-transduced OCs, assessed by qRT-PCR shown as fold change over LV-oe*Ckap4-*Ctrl group (*n* = 3). (K) Immunoblotting of CKAP4 protein in LV-oe*Ckap4-*Ctrl- and LV-oe*Ckap4*-transduced OCs. (L) TRAP staining of LV-oe*Ckap4-*Ctrl- and LV-oe*Ckap4*-transduced OCs at 0 h, 96 h, and STS + 4 h. (M–N) Quantification of OC number per mm^2^(M) and TRAP⁺ area percentage (N) in LV-oe*Ckap4-*Ctrl- and LV-oe*Ckap4*-transduced OCs.

Results are shown as mean ± SD. Each dot indicates an individual sample. Student’s t test (A-N) was performed. ****P* < 0.001; ***P* < 0.01; **P* < 0.05; ns, not significant.


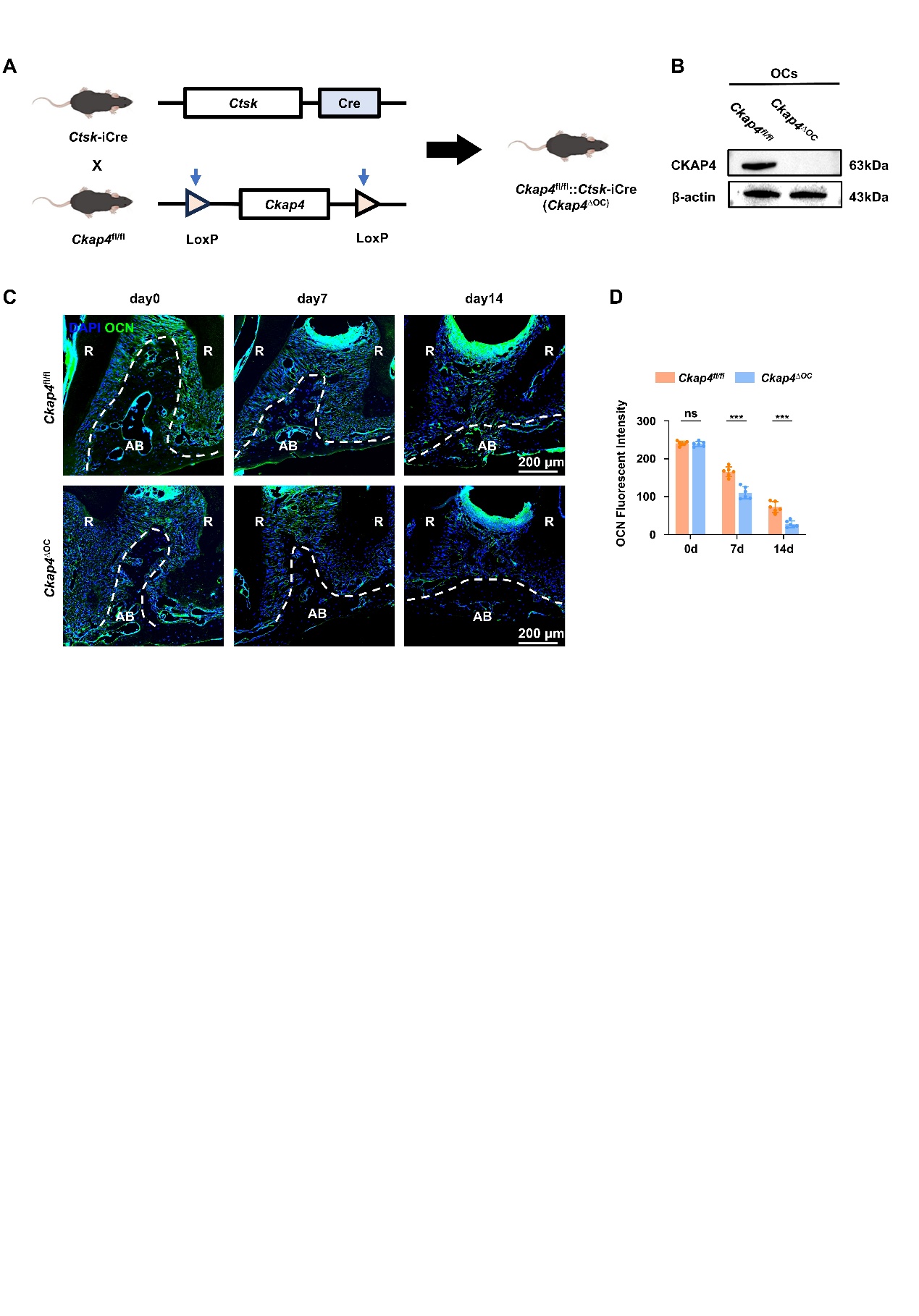


**Figure S3 | Generation of OC-conditional *Ckap4* knockout mice and OCN expression analysis.**

(A) Schematic of generating *Ckap4^ΔOC^* mice: *Ctsk*-Cre mice were crossed with *Ckap4^fl/fl^* mice to obtain *Ckap4^ΔOC^* mice. (B) Immunoblotting of CKAP4 protein in OCs from *Ckap4^fl/fl^* and *Ckap4^ΔOC^* mice, with β-actin as a loading control. (C) Immunofluorescence staining of OCN (green) in maxillary sections from *Ckap4^fl/fl^* and *Ckap4^ΔOC^* mice at 0, 7, and 14 days (nuclei stained with DAPI, blue; dashed lines outline alveolar bone). (D) Quantification of OCN fluorescent intensity in maxillary sections from *Ckap4^fl/fl^* and *Ckap4^ΔOC^* mice at indicated time points (*n* = 3).

Results are shown as mean ± SD. Each dot indicates an individual sample or mouse. Student’s t test (D) was performed. ****P* < 0.001; ***P* < 0.01; **P* < 0.05; ns, not significant.


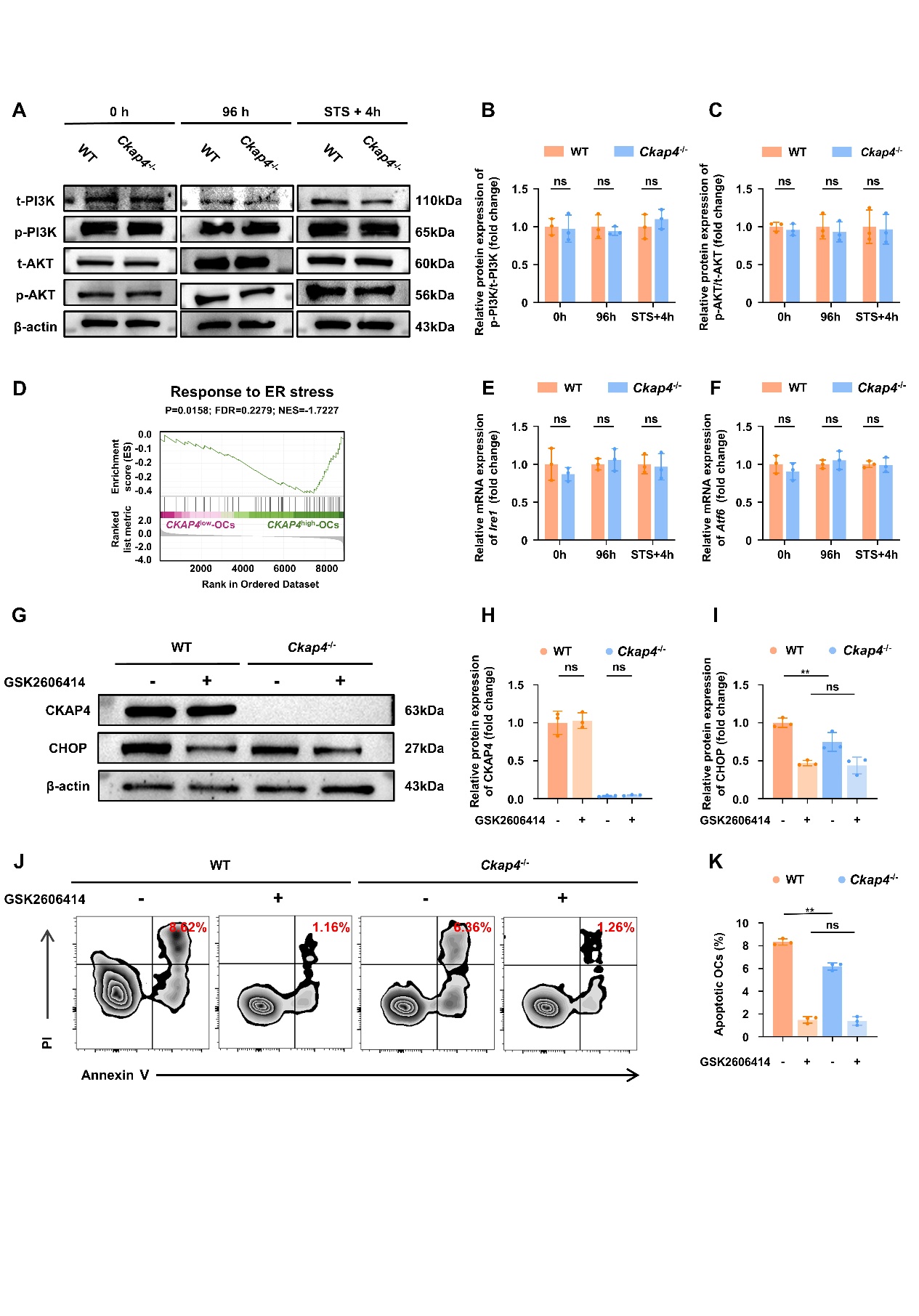


**Figure S4 | PI3K-AKT pathway is not involved in CKAP4-mediated OC apoptosis, and PERK is involved in the regulation of CHOP by CKAP4.**

(A) Immunoblotting of t-PI3K, p-PI3K, t-AKT, p-AKT, and β-actin (loading control) in WT and *CKAP4^-/-^* OCs at 0 h, 96 h, and STS + 4 h. (B–C) Quantification of p-PI3K/t-PI3K (B), p-AKT/ t-AKT (C) expression in WT and *CKAP4^-/-^* OCs at 0 h, 96 h, and STS + 4 h shown as fold change over WT group (*n* = 3). (D) Enrichment results for Response to ER stress pathways via GSEA. (E) and (F) *Ire1* and *Atf6* expression in WT and *CKAP4^-/-^* OCs at 0 h, 96 h, and STS + 4 h was detected by RT-qPCR, normalized to *β-actin*, and shown as fold change over WT group (*n* = 3). (G) Immunoblotting of CKAP4, CHOP, and β-actin (loading control) in WT and *Ckap4^-/-^* OCs with/without GSK2606414 (PERK inhibitor) treatment. (H-I) Quantification of CKAP4 (H) and CHOP (I) protein levels in WT and *Ckap4^-/-^* OCs with/without GSK2606414 treatment from panel g shown as fold change over WT without GSK2606414 treatment group (*n* = 3). (J) and (K) Flow cytometry analysis (J) and quantification (K) of apoptotic OCs (Annexin V⁺/PI⁺) in WT and *Ckap4^-/-^* OCs with/without GSK2606414 treatment (*n* = 3).

Results are shown as mean ± SD. Each dot indicates an individual sample. Student’s t test and (B-F) one-way ANOVA with Tukey’s post hoc test (H-K) were performed. ****P* < 0.001; ***P* < 0.01; **P* < 0.05; ns, not significant.


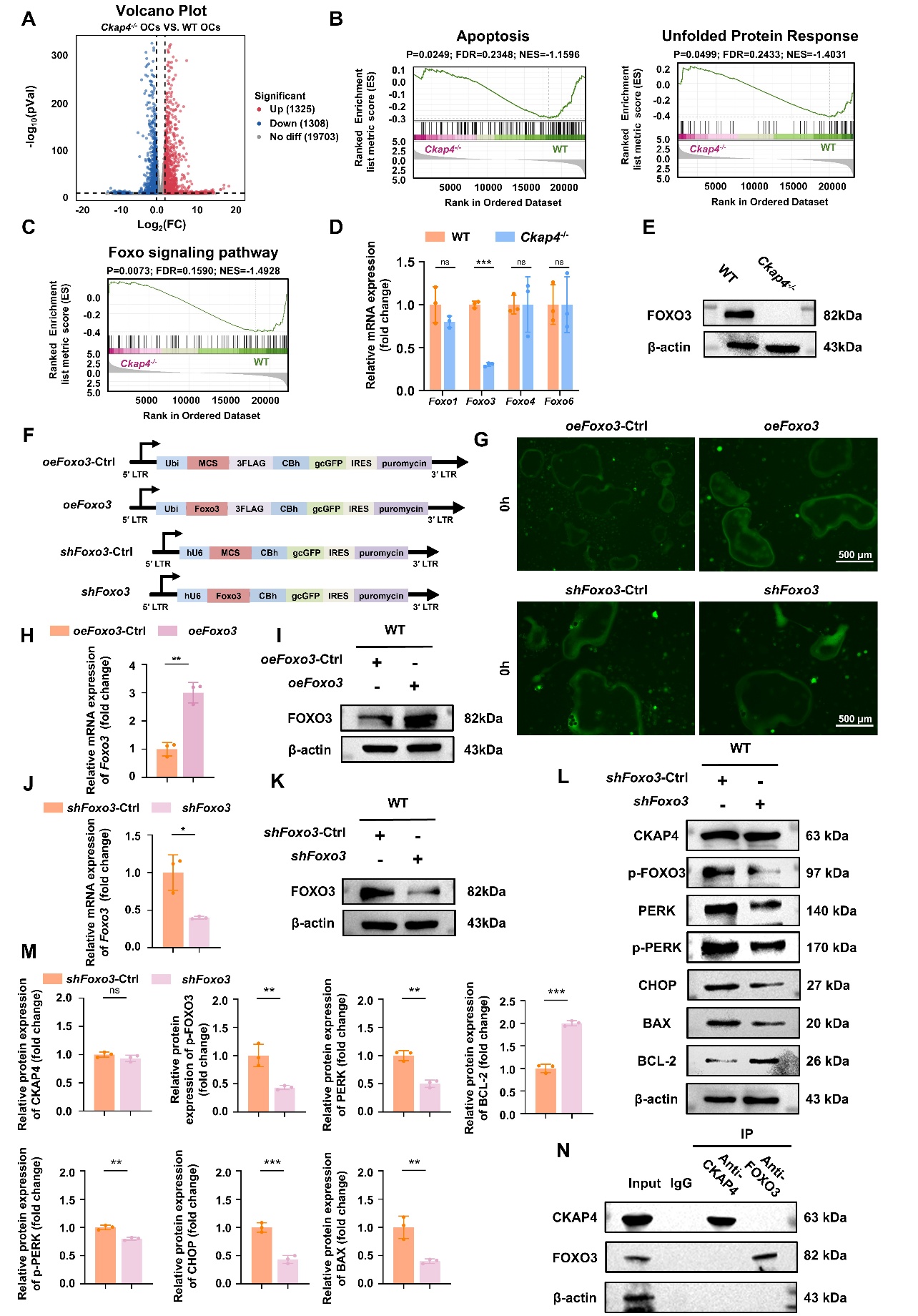


**Figure S5 | Transcriptomic analysis and FOXO3 viral manipulation validation.**

(A) Volcano plot of DEGs between WT and *Ckap4*^-/-^ OCs. (B) and (C) Enrichment results for OC apoptosis (B), UPR pathways (B) and Foxo signaling pathway (C) via GSEA. (D) *Foxo1*, *Foxo3*, *Foxo4* and *Foxo6* expression during natural apoptosis was detected by RT-qPCR, normalized to *β-actin*, and shown as fold change over WT group (*n* = 3). (E) The expression levels of FOXO3 in OCs during natural apoptosis were confirmed by WB assays. β-actin was used as a loading control. (F) Schematic of lentiviral vectors: *oeFoxo3*-Ctrl or *oeFoxo3* for overexpressing *Foxo3*, and *shFoxo3*-Ctrl or *shFoxo3* for silencing *Foxo3*, all with gcGFP. (G) Microscopy images of OCs transfected with LVs from panel F. (H) *Foxo3* mRNA expression in WT OCs with LV-oe*Foxo3*-Ctrl and LV-*oeFoxo3* treatment, measured by qRT-PCR shown as fold change over oe*Foxo3*-Ctrl group (*n* = 3). (I) Immunoblotting of FOXO3 protein levels in WT OCs with LV-oe*Foxo3*-Ctrl and LV-*oeFoxo3* treatment. (J) *Foxo3* mRNA expression in WT OCs with LV-sh*Foxo3*-Ctrl and LV-*shFoxo3* treatment, measured by qRT-PCR shown as fold change over sh*Foxo3*-Ctrl group (*n* = 3). (K) Immunoblotting of FOXO3 protein levels in WT OCs with LV-sh*Foxo3*-Ctrl and LV-*shFoxo3* treatment. (L) Immunoblotting of CKAP4, p-FOXO3, PERK, p-PERK, CHOP, BAX, BCL-2 and β-actin (loading control) in WT and *Ckap4*^-/-^ OCs with LV-*shFoxo3* or LV-*shFoxo3*-Ctrl treatment. (M) Quantification of protein levels from panel f. Expression was detected by WB assay, normalized to β-actin, and presented as fold change relative to WT OCs with LV-*shFoxo3*-Ctrl group (*n* = 3). (N) Co-IP analysis of CKAP4 and FOXO3.

Results are shown as mean ± SD. Each dot indicates an individual sample. Student’s t test and (D-M) was performed. ****P* < 0.001; ***P* < 0.01; **P* < 0.05; ns, not significant.


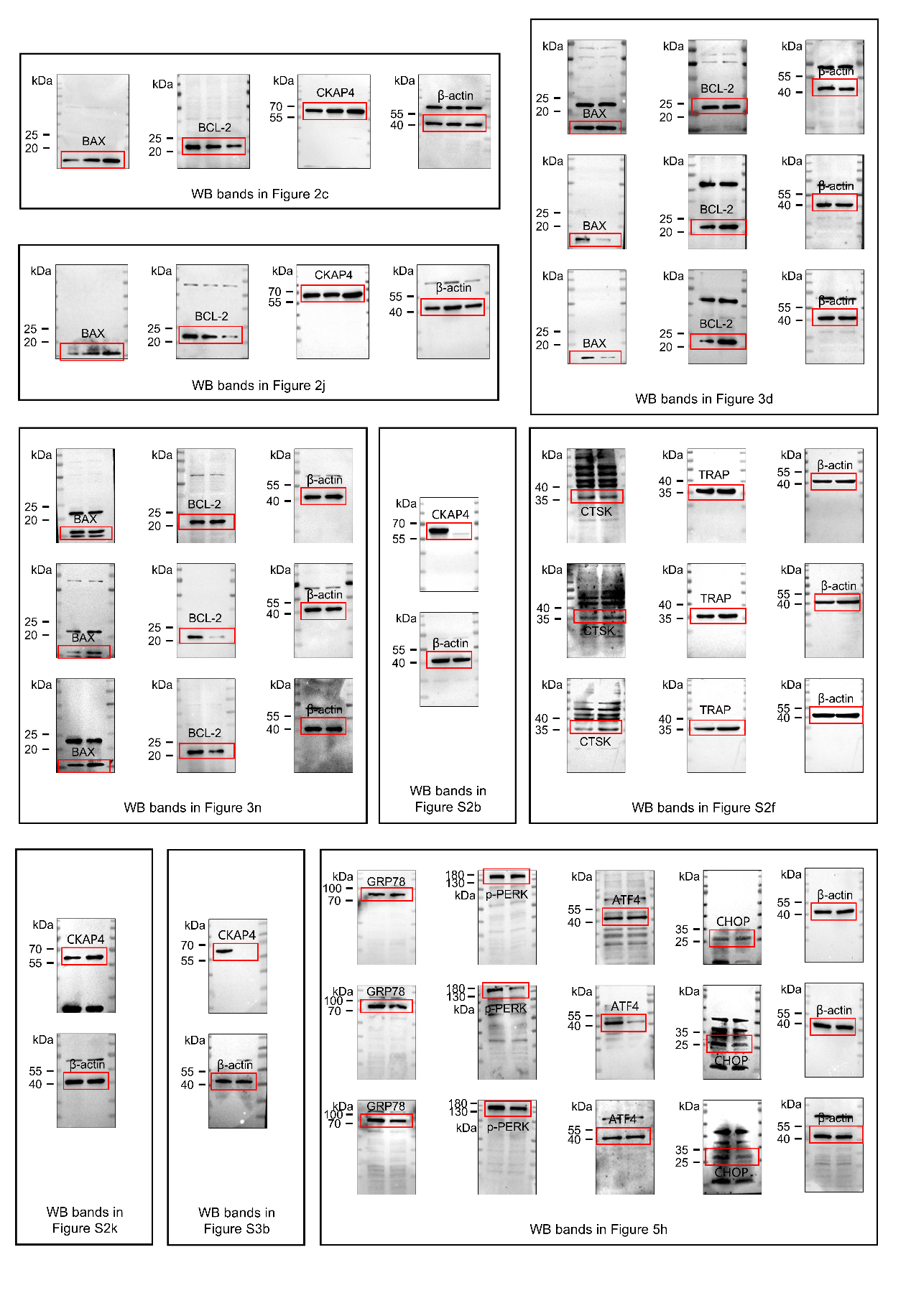


**Figure S6 | Uncropped pictures of the western blot bands presented in this study.**

**
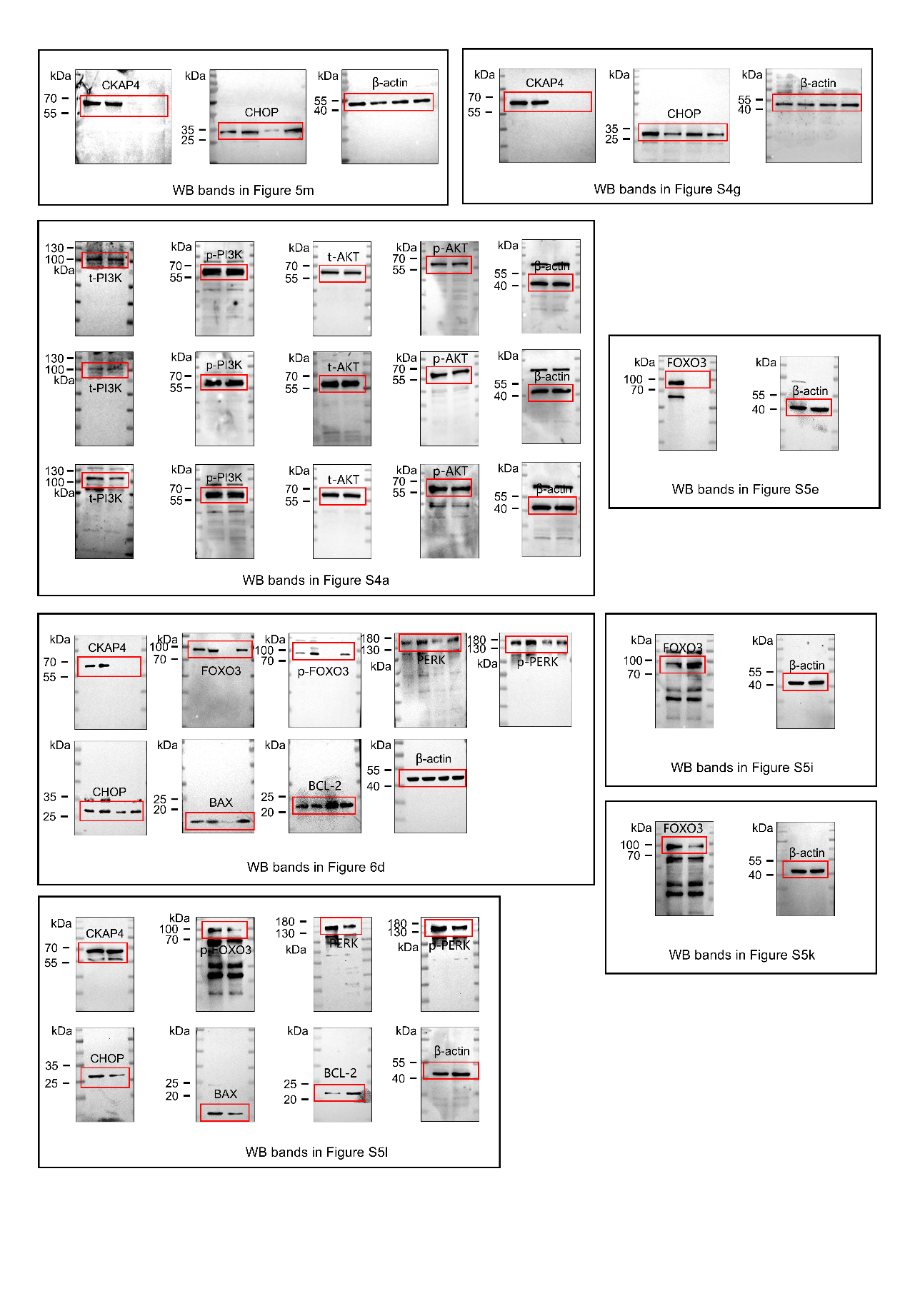
**

**Figure S7 | Uncropped pictures of the western blot bands presented in this study.**

**Table S1 | Oligonucleotides used for genotyping**

| **Genes** | **Orientation** | **Sequence (5′→3′)** |
| --- | --- | --- |
| *Ckap4^-/-^* | Forward | TTATTGTCTCGGTTCTTAGGCGTT |
|  | Reverse | ATGGAGAACAATTACAGAAACGCAG |
| *Ckap4^fl/fl^* | Forward | GTTTCCGTGTGCTGTCTTGATTGA |
|  | Reverse | AAGGTAGTTTTACGTGGTGGCTGTG |
| *Ctsk*-iCre | Forward | TGATCTATGGTGCCAAGGATGACT |
|  | Reverse | CAGAGAGAAGGGAAGTAGAGTTGT |

**Table S2 | Antibodies used for WB**

| **Antibody** | **Source** | **CAT#** | **RRID** |
| --- | --- | --- | --- |
| Bax Antibody | Cell Signaling Technology | 2772 | AB_10695870 |
| Bcl-2 (D17C4) Rabbit mAb | Cell Signaling Technology | 3498 | AB_1903907 |
| Anti-CKAP4 antibody | Abcam | ab302539 |  |
| Cathepsin K (CTSK) Rabbit Polyclonal Antibody | OriGene | TA375221 |  |
| TRAP (D-3) | Santa Cruz Biotechnolog | sc-376875 | AB_3075340 |
| Anti-GRP78 BiP antibody | Abcam | ab21685 | AB_2119834 |
| PERK (C33E10) Rabbit mAb | Cell Signaling Technology | 3192 | AB_2095847 |
| ATF-4 (D4B8) Rabbit mAb | Cell Signaling Technology | 11815 | AB_2616025 |
| CHOP (L63F7) Mouse mAb | Cell Signaling Technology | 2895 | AB_2089254 |
| Anti-AKT1 + AKT2 + AKT3 antibody | Abcam | ab179463 | AB_2810977 |
| Phospho-AKT (Ser473) Mouse anti-Human, Mouse | proteintech | 66444-1 | AB_2782958 |
| PI3 Kinase p110α (C73F8) Rabbit mAb | Cell Signaling Technology | 4249 | AB_2165248 |
| Phospho-PI3 Kinase p85 (Tyr458)/p55 (Tyr199) Antibody | Cell Signaling Technology | 4228 | AB_659940 |
| FoxO3a (D19A7) Rabbit mAb | Cell Signaling Technology | 12829 | AB_2636990 |
| FoxO1 (C29H4) Rabbit mAb | Cell Signaling Technology | 2880 | AB_2106495 |
| Phospho-FoxO3a (Ser253) Antibody | Cell Signaling Technology | 9466 | AB_2106674 |
| Anti β-Actin Mouse Monoclonal Antibody | CWBio | CW0096 | AB_2665433 |

**Table S3 | Oligonucleotides used for RT-qPCR**

| **Genes** | **Orientation** | **Sequence (5′→3′)** |
| --- | --- | --- |
| *Bax* | Forward | AAGCGACTGATGTCCCTGTCTC |
|  | Reverse | GATGGTGAGTGAGGCGGTGAG |
| *Bcl-2* | Forward | GACTTCGCCGAGATGTCCAG |
|  | Reverse | GAACTCAAAGAAGGCCACAATC |
| *Ckap4* | Forward | CGTCCCTGGAGAACACAGTC |
|  | Reverse | AATGTCCCTCTCCCTCGACA |
| *β-actin* | Forward | CACGATGGAGGGGCCGGACTCATC |
|  | Reverse | TAAAGACCTCTATGCCAACACAGT |
| *Grp78* | Forward | TCAAGTTCTTGCCGTTCAAGG |
|  | Reverse | AAATAAGCCTCAGCGGTTTCTT |
| *Perk* | Forward | GTGATAAAGGTTTCGGTTGCTG |
|  | Reverse | TGTTTTCTGTGGCTCCTCTGG |
| *Atf4* | Forward | CATGGGTTCTCCAGCGACA |
|  | Reverse | TCTGGCATGGTTTCCAGGTC |
| *Chop* | Forward | CAAGAGGTCCTGTCTTCAGATGA |
|  | Reverse | TCTGTTTCCGTTTCCTGGTTC |
| *Ire1α* | Forward | CCTAGTCAGTTCTGCGTCCG |
|  | Reverse | TTCCATCCAGCGTTGACACA |
| *Atf6* | Forward | CGGAGTATTTTGTCCGCCTG |
|  | Reverse | GCTGCTTCCAATTGCAGCTC |
| *FoxO1* | Forward | AACCTGGCATTACAGTTGGCC |
|  | Reverse | AAATGCAGGAGGCATGACTACGT |
| *FoxO3* | Forward | AAGCCAGCTACCTTCTCTTCCA |
|  | Reverse | GTGGCTAAGTGAGTCCGAAGTGA |
| *FoxO4* | Forward | AGGCCACCGGCAAAAGCTCTT |
|  | Reverse | CTTCCGTCCACGAAGCAG |
| *FoxO6* | Forward | CTGGCAAGAGTTCATGGTGG |
|  | Reverse | GTGCAGCTGCTTCTTCTTGC |

**Table S4 | Oligonucleotides of LV**

| **Genes** | **Sequence (5′→3′)** |
| --- | --- |
| oe*Ckap4* | (Transcript: NM_175451.1) |
| *oeFoxo3*^-^ | (Transcript: NM_175451.1) |
| *LV-Foxo3-RNAi(PSC130782-1)* | GGAGTTTGGTCAATCAGAACT |
| *LV-Foxo3-RNAi(PSC130783-1)* | GGCTCACTTTGTCCCAGATCT |
| *LV-Foxo3-RNAi(PSC130784-1)* | GGAGCTTGGAATGTGACATGG |
| *LV-shFoxo3-Ctrl* | TTCTCCGAACGTGTCACGT |
